# Sympathovagal crosstalk: Y2-receptor blockade enhances vagal effects which in turn reduce NPY levels via muscarinic receptor activation

**DOI:** 10.1093/cvr/cvaf180

**Published:** 2025-10-06

**Authors:** Neil R Jani, Valerie Y H van Weperen, Thamali Ayagama, Jonathan D Hoang, Maryam Emamimeybodi, Benjamin Mothibe Bussmann, Sartaj Bal, Ashna Kumar, Artin Khaky, David Hamon, Neil Herring, Corey Smith, Marmar Vaseghi

**Affiliations:** Cardiac Electrophysiology Programs, Division of Cardiology, Department of Medicine, University of California, 100 Medical Plaza, Suite 660, Los Angeles, CA 90095, USA; Cardiac Electrophysiology Programs, Division of Cardiology, Department of Medicine, University of California, 100 Medical Plaza, Suite 660, Los Angeles, CA 90095, USA; Burdon Sanderson Cardiac Science Centre, Department of Physiology, Anatomy and Genetics, University of Oxford, Oxford, UK; Cardiac Electrophysiology Programs, Division of Cardiology, Department of Medicine, University of California, 100 Medical Plaza, Suite 660, Los Angeles, CA 90095, USA; Cardiac Electrophysiology Programs, Division of Cardiology, Department of Medicine, University of California, 100 Medical Plaza, Suite 660, Los Angeles, CA 90095, USA; Burdon Sanderson Cardiac Science Centre, Department of Physiology, Anatomy and Genetics, University of Oxford, Oxford, UK; Cardiac Electrophysiology Programs, Division of Cardiology, Department of Medicine, University of California, 100 Medical Plaza, Suite 660, Los Angeles, CA 90095, USA; Cardiac Electrophysiology Programs, Division of Cardiology, Department of Medicine, University of California, 100 Medical Plaza, Suite 660, Los Angeles, CA 90095, USA; Cardiac Electrophysiology Programs, Division of Cardiology, Department of Medicine, University of California, 100 Medical Plaza, Suite 660, Los Angeles, CA 90095, USA; Division of Cardiology, Department of Medicine, University of Iowa, Iowa City, IA, USA; Burdon Sanderson Cardiac Science Centre, Department of Physiology, Anatomy and Genetics, University of Oxford, Oxford, UK; Department of Physiology and Biophysics, Case Western Reserve University, Cleveland, OH, USA; Cardiac Electrophysiology Programs, Division of Cardiology, Department of Medicine, University of California, 100 Medical Plaza, Suite 660, Los Angeles, CA 90095, USA

**Keywords:** Neuropeptide Y, Vagus nerve stimulation, Y2 receptor, Sympathetic, Muscarinic receptors, Cardiac autonomic nervous system

## Abstract

**Aims:**

Ventricular arrhythmias are associated with sympathoexcitation and increased co-transmitter neuropeptide Y (NPY) levels. Vagal nerve stimulation (VNS) has been reported to decrease release of norepinephrine, while NPY has been reported to decrease acetylcholine release *ex vivo* by binding Y2 receptors on parasympathetic nerves. We hypothesized that VNS reduces NPY levels via a muscarinic receptor (MR) mediated mechanism *in vivo* and that, in turn, blockade of pre-synaptic Y2R can further enhance the effects of VNS and decrease the effects of sympathoexcitation by increasing vagal tone.

**Methods and results:**

Single-cell RNA sequencing of rat stellate ganglia and immunohistochemistry were performed and identified the M2 receptor as the predominant subtype on NPY-expressing sympathetic neurons. *Ex vivo* field stimulation of rat stellate ganglia, before and after application of carbamylcholine (CCH; muscarinic agonist) and atropine (muscarinic blocker) showed that CCH reduced NPY release, while the addition of atropine increased NPY levels. Subsequently, to validate *ex vivo* findings, *in vivo* effects of VNS during bilateral stellate ganglia stimulation (BSS) on NPY release with and without atropine were evaluated and haemodynamic and electrophysiological parameters, including ventricular activation recovery intervals (ARIs, a surrogate for action potential duration), and real-time *in vivo* interstitial NPY levels were measured. Post-atropine, suppression of NPY by VNS was significantly diminished, confirming a MR mediated mechanism *in vivo*. Finally, in a porcine model *in vivo*, effects of VNS on NPY levels and of the Y2R blocker, BIIE0246, during BSS were tested. These studies demonstrated that Y2R blockade significantly reduced the cardiac effects of BSS on systolic pressure, inotropy, and ARIs. While the ventricular effects of VNS, including suppression of interstitial NPY levels, haemodynamic, and electrophysiological parameters were enhanced by Y2R blockade, heart rate remained unaffected.

**Conclusion:**

Vagal activation reduces interstitial NPY levels via a pre-synaptic sympathetic M2R mechanism. Y2R inhibition reduces effects of sympathoexcitation and enhances the effects of VNS *in vivo*. These findings highlight the role of NPY in sympathovagal crosstalk and suggest Y2R as a potential target to modulate autonomic balance.

## Graphical Abstract

**Graphical Abstract:**
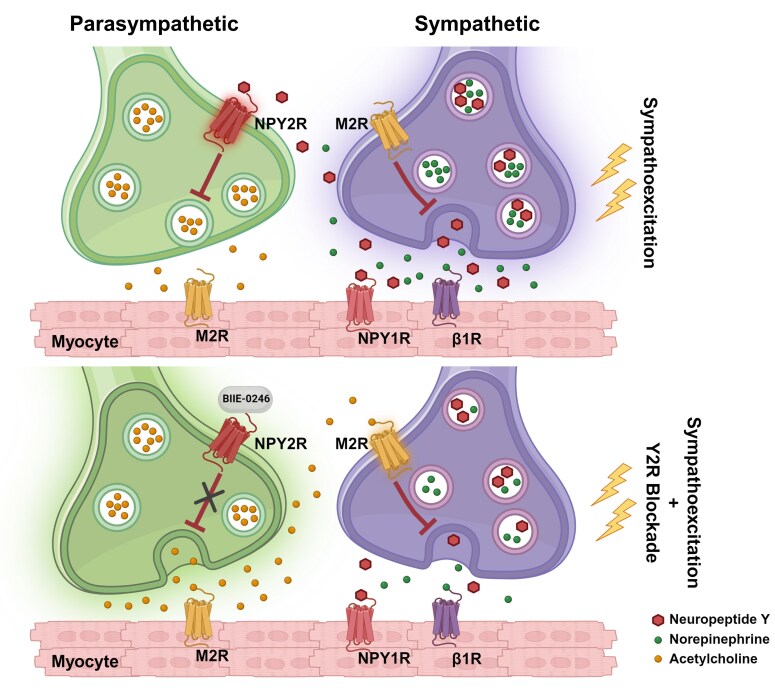

**Time of primary review: 78 days**

## 1. Introduction

Myocardial injury leads to pathological autonomic remodelling, characterized by sympathetic activation and vagal dysfunction.^1,2^ This autonomic imbalance has been demonstrated to increase the risk of ventricular arrhythmias, sudden cardiac death, and development and progression of heart failure.^3–5^ During periods of significant sympathoexcitation, the sympathetic co-transmitter neuropeptide Y (NPY) is co-released with norepinephrine from sympathetic nerve terminals.^6–8^ Elevated NPY levels have been shown to increase mortality, both following myocardial infarction^9,10^ and in the setting of heart failure.^11^ In addition, NPY has been shown to have direct, pro-arrhythmic electrophysiological effects, independent of norepinephrine.^12^ These effects have been shown to be mediated through Y1 receptors on cardiomyocytes, and elevated NPY levels increase the susceptibility to ventricular arrhythmias.^13^ On the other hand, healthy vagal tone plays a pivotal role in counteracting sympathetic overactivity. Vagal activation releases acetylcholine,^14^ which suppresses norepinephrine levels^15^ and has been suggested to have anti-arrhythmic effects.^16^ Notably, in *ex vivo* atrial tissue, NPY has been reported to reduce acetylcholine release by activation of pre-synaptic Y2 receptors (Y2R), potentially limiting the effects of vagal activation.^17^ However, *in vivo* data on whether Y2R blockade enhances acetylcholine release and the resultant electrophysiological effects of vagal nerve stimulation (VNS) are lacking. As NPY has a much longer half-life than norepinephrine,^18,19^ it can have more detrimental and longer-lasting electrophysiological consequences. In this study, we hypothesized that VNS could decrease the release of NPY from sympathetic ganglia via a muscarinic receptor (MR) mediated mechanism. We further hypothesized that blocking Y2R may mitigate cardiac sympathetic effects by improving the underlying vagal tone (i.e. usually suppressed by NPY), thereby enhancing the haemodynamic and electrophysiological effects of VNS. We first performed single-cell RNA sequencing (scRNAseq) and immunohistochemical analysis of rat stellate ganglia to evaluate for the presence and subtype of MRs on NPY-expressing stellate ganglia neurons and assessed the *ex vivo* effects of MR activation and blockade on NPY levels. Based on these findings, we evaluated the effects of MR blockade on NPY release *in vivo* in the porcine model. Finally, to evaluate sympathovagal crosstalk and the role of Y2R blockade, intermittent VNS during continuous sympathetic stimulation (to elevate NPY levels) before and after infusion of a Y2R antagonist was performed to determine whether Y2R inhibition could enhance the effects of VNS on ventricular electrophysiological and haemodynamic parameters *in vivo* and further suppress NPY levels.

## 2. Methods

### 2.1 Ethical approval

The rodent studies were approved by the University of Oxford's Animal Ethics Committee and conducted in compliance with the guidelines of the Animals (Scientific Procedures) Act 1986 (UK) and performed under British Home Office Project License PP1225128.

All porcine experiments were approved by the University of California, Los Angeles Institutional Animal Care and Use Committee and performed in accordance with the National Institutes of Health Guide for the Care and Use of Laboratory Animals.

### 2.2 Rodent studies

#### 2.2.1 Single-cell RNA sequencing of rat stellate ganglia

Four- to 6-week-old male Wistar rats (*n* = 4) were sacrificed with an overdose of anaesthetic (pentobarbital 0.3 mL/100 g, under 5% isoflurane and 95% oxygen) and cervical dislocation according to Animals (Scientific Procedures) Act schedule 1. Single-cell suspensions of right and left stellate ganglia were prepared via enzymatic and mechanical dissociation as described previously.^20^ ScRNAseq was performed by the Wellcome Trust Centre for Human Genetics via 10× Genomics Chromium (10× Genomics, USA) (Single Cell 3' v3) and Illumina HiSeq 4000 (Illumina, USA). The dataset is available at Genome Expression Omnibus (GSE144027). Data were aligned using the Cell Ranger 10× genomics pipeline (V3.0.3) (Rnor6.0) with default parameters and exported to Seurat (v5.0). Cells were excluded in Seurat if the number of counts per cell was more than 4000 or less than 200 and if the percentage of mitochondrial genes was more than 30%. SCTransform was used to scale and normalize data using default parameters and regress out percentage of mitochondrial genes and number of counts per cell. Dimensionality reduction was performed with principal component analysis using 30 principal components. Clustering was performed with FindNeighbors (30 dimensions) and FindClusters (resolution of 0.6). UMAP (30 dimensions) was used to further reduce dimensionality to two dimensions for visualization. Differential expression analysis was performed via Wilcoxon rank sum test within Seurat to determine marker genes to identify each cluster. Neuronal cells were identified by Snap25, Syt1, Pcsk1n, and Prph.^21^

#### 2.2.2 Immunohistochemistry

Four-week-old male Wistar rats (*n* = 4) were sacrificed as above and the right and left stellate ganglia dissected, desheathed, and enzymatically digested. After a sequential mechanical trituration, cell suspension containing stellate neurons was plated onto poly-d/lysine/laminin-coated coverslips and cultured for 48 h as described previously.^20,22^ Cells were fixed for 10 min in 4% paraformaldehyde, followed by three 30 s washes in phosphate-buffered saline and then blocked and permeabilized in 0.1% Triton-X-100, with donkey serum and 3% BSA for 30 min. Cells were subsequently washed three more times in phosphate-buffered saline. Cells were co-labelled with mouse anti-Chrm2 (MA3044, Invitrogen) and sheep anti-TH (PA1–4679, Invitrogen) primary antibodies for a period of 1 h at 37°C. Cells were washed three times in phosphate-buffered saline before anti-mouse Alexa Fluor 568 (A21124, Invitrogen) and anti-sheep Alexa Fluor 647 (A21448, Invitrogen) were used as secondary antibodies. Cells were again washed three times in phosphate-buffered saline before cell nuclei counterstaining with Hoechst 33342 stain for 1 min and washed three more times in phosphate-buffered saline before images were acquired at 40× magnification on an inverted Nikon microscope.

#### 2.2.3 NPY release from isolated rat stellate ganglia

Adult male Wistar rats (*n* = 32) were sacrificed as above, and the right and left stellate ganglia dissected. Two pairs of stellates (right and left from two rats) were transferred to a 1 mL static water jacketed organ bath (36–37°C). After an equilibration period of 20 min, the perfusate was replaced and stellates field stimulated at 10 Hz for 2 min (1 ms, 25 V)^13,23,24^ with the perfusate replaced immediately afterwards. The stellates were then incubated with carbamylcholine (CCH, 200 nM, *n* = 18 rats, nine experiments) or carbamylcholine and atropine (CCH + atropine; 1 mM, *n* = 14 rats, seven experiments) for 20 min,^24^ before replacing the perfusate and repeating field stimulation at 10 Hz for 2 min (1 ms, 25 V) with the perfusate again replaced after stimulation. Perfusate samples were analysed using enzyme-linked immunosorbent assay (EZHNPY-25K, Millipore, USA) according to manufacturer’s instructions, to detect the level of NPY release from each stimulation. The perfusate solution contained (mmol/L) NaCl 120, KCl 4, MgSO_4_*7H_2_O 1.3, NaH_2_PO_4_*2H_2_O 1.2, CaCl_2_ 1.2, NaHCO_3_ 25.2, Glucose 11, and was constantly aerated with carbogen (95% O_2_, 5% CO_2_), to maintain a pH of 7.35–7.45.

### 2.3 Porcine studies

#### 2.3.1 Experimental protocol

A total of 13 male Yorkshire pigs (S&S Farms; 3.5 ± 0.2 months old; 46.2 ± 1.3 kg, *n* = 13) were used in this study. Pigs were quarantined for a minimum of 1 week upon arrival for acclimatization and were subjected to a standard 12-h light/12-h dark cycle. Five animals underwent BSS with intermittent VNS (BSS + VNS) before and after muscarinic blockade with atropine. Eight animals underwent continuous bilateral stellate ganglia stimulations (BSS) with intermittent VNS (BSS + VNS) pre- and post-Y2R blockade with BIIE0246 (BIIE).

#### 2.3.2 Surgical preparation

All animals were sedated with tiletamine-zolazepam (4–8 mg/kg, I.M.) and intubated. General anaesthesia was maintained with isoflurane (1–2%) and analgesia managed by infusion of one dose of fentanyl (20 mcg/kg, I.V.) during surgical preparation. Following the completion of surgical procedures, anaesthesia was switched to α-chloralose (Sigma-Aldrich, 50 mg/kg initial bolus followed by 20–30 mg/kg/h continuous infusion). Hourly arterial blood gases were monitored, and ventilator adjustments were made to maintain acid–base homeostasis. Core body temperature was assessed using a rectal probe, and heating pad temperatures were adjusted to maintain body temperature between 35 and 38°C. Bilateral femoral veins and arteries were accessed percutaneously, and sheaths inserted in the femoral veins for continuous saline and drug infusion and in the femoral arteries for Millar pressure catheter placement and invasive blood pressure monitoring. Bilateral incisions were made 1 cm lateral to the midline, at the level of the cricoid cartilage, to expose the vagus nerve within the carotid sheath. Median sternotomy was performed to expose the heart. The right and left stellate ganglia were isolated behind the parietal pleura at the level of the C7-T1 intercostal space. After the completion of experiments, animals were euthanized under deep anaesthesia by induction of ventricular fibrillation (VF) via application of direct electrical current.

#### 2.3.3 Haemodynamic assessments

Left ventricular (LV) pressure was continuously monitored using a 5 Fr pressure-conductance catheter (Millar SPR-350, Millar, Inc., Pearland, TX, USA). Raw signals were digitized and recorded by a CED Power1401, and analysed using Spike2 software (Cambridge Electronic Design, UK). Twelve-lead surface electrocardiograms (ECGs) were obtained via a GE CardioLab System (GE Healthcare, Fairfield, CT, USA), with precordial leads placed on the dorsum of the animal at the mid-thoracic level, due to the midline sternotomy.

#### 2.3.4 Vagal nerve stimulation

Bipolar cuff electrodes were placed around the isolated left and right cervical vagi. For standardization of stimulations across animals, the threshold current was defined as the current which decreased heart rate (HR) by 10% (10 Hz, 1 ms). VNS was performed at 1.2× this current.^25^ Bilateral intermittent VNS (10 s ON, 50 s OFF, 10 Hz, 1 ms)^25–27^ was performed during the 3 min of continuous BSS. VNS parameters were selected in accordance with prior studies showing that these are sufficient to capture vagal cardiomotor nerve fibres and lead to haemodynamic and ventricular electrophysiological effects without causing heart block.^26,28^ The frequency of VNS was also selected to avoid atrial fibrillation and significant atrial effective refractory period shortening, which have been observed with higher frequencies of stimulation.^29^ These individualized settings also ensured that stimulation effects were consistent across animals (given that they can otherwise vary from animal to animal depending on the electrode micro-environment). Electrophysiological and haemodynamic variables, and real-time ventricular interstitial NPY levels were measured before and during BSS + VNS, pre- and post-atropine and pre- and post-BIIE. The same stimulation parameters were used before and after administering atropine and BIIE.

#### 2.3.5 Stellate ganglia stimulations

Bipolar cuff electrodes (MicroProbes, Gaithersburg, MD, USA) were placed around the ganglia and connected to a Grass S88 stimulator for BSS.^30,31^ To standardize stimulations across animals, a threshold stimulation current was determined for each stellate ganglion, as the degree/effects of stimulation can be influenced by individual electrode impedance and the tissue micro-environment. The threshold stimulation current was determined for each animal as the current that induced a 10% increase in HR or systolic blood pressure at 4 Hz, 4 ms pulse-width (square wave).^32^ BSS was then performed at this threshold in each animal at 10 Hz, 4 ms for a minimum of 3 min, pre- and post-administration of atropine or BIIE. We have previously shown that these sympathetic stimulation parameters lead to the release of norepinephrine and NPY and have significant haemodynamic and ventricular electrophysiological effects.^12^ Further increases in NPY levels were not observed with the use of higher frequencies of stimulation in the porcine model.^12^ A minimum of 60 min was allowed for electrophysiological and haemodynamic parameters to return to baseline between stimulations. Electrophysiological, haemodynamic variables, and real-time interstitial LV myocardial NPY levels were measured and analysed before and during stimulations.

#### 2.3.6 Cardiac electrophysiological measurements

Unipolar epicardial electrograms were obtained via a 56-electrode sock placed over the ventricles, connected to a GE CardioLab Recording System (GE Healthcare, Chicago, IL, USA), band pass filtered at 0.05–500 Hz, and mapped onto 2D-polar maps. Global activation recovery intervals (ARIs), a surrogate of local action potential duration,^33,34^ were analysed using customized software, iScaldyn (University of Utah, Salt Lake, UT, USA), as previously described.^16,30^ Activation time (AT) was defined as the interval from the onset of unipolar epicardial electrograms to the minimal d*V*/d*t* of the depolarization wave-front; and repolarization time (RT) from the onset of the electrogram to the maximal dV/dt of the repolarization wave-front. ARI was calculated by subtracting AT from RT.^33,34^ ARIs have been shown to be an accurate measurement of local action potential durations, including during adrenergic interventions.^33^

#### 2.3.7 Atropine infusion

Atropine was administered (0.04 mg/kg bolus, I.V.)^25^ in pigs (*n* = 5) to determine whether the effects of VNS on interstitial ventricular NPY levels were mediated by MRs. Bilateral intermittent VNS (10 s ON, 50 s OFF) was performed at 1.2× threshold current (10 Hz, 1 ms) during BSS. BSS was performed continuously for 3 min, as described above, to raise NPY levels and simulate a state of sympathoexcitation. Before and after atropine administration, ARIs, haemodynamic variables, and ventricular interstitial NPY levels were measured before and during BSS with intermittent VNS.

#### 2.3.8 Infusion of Y2R blocker

In a separate group of animals (*n* = 8), the Y2R blocker, BIIE0246 (BIIE, Sigma-Aldrich, St. Louis, MO, USA), was infused intravenously (32 nmol/kg bolus followed by a 2 nmol/kg/min infusion). A 20-min acclimation period with continuous infusion of BIIE was allowed post-bolus completion and prior to any repeat autonomic stimulations (intermittent VNS during continuous sympathetic stimulations) to allow for stabilization of pharmacologic effects.

#### 2.3.9 Measurement and analysis of cardiac interstitial NPY levels using capacitive immunoprobes

Capacitive immunoprobes (CI) were used to measure real-time, local, ventricular interstitial NPY levels.^35,36^ Advantages of CI include its ability to continuously measure interstitial NPY profiles *in vivo* and allow for local measurements that better represent these levels by overcoming the dilutionary effects of coronary sinus plasma measurements. CI probes were generated using electrodeposition of dopamine on the distal portion of platinum wires (coated diameter of 0.005′, A-M Systems, Sequim, WA, USA) by immersion of wires in a 5 mM dopamine solution. Probe potential was ramped between −0.6 and +0.65 V at 0.4 V/s for 420 s.^35^ The dopamine-deposited platinum electrodes were then soaked in a solution containing NPY antibody (ab112473, Abcam, Cambridge, MA, USA) for 1 h, to allow binding of antibodies onto the probes. The proximal portion of the wires were stripped of their perfluoroalkoxy coating and crimped into a 1 mm gold plated connector pin. Calibration of CI is performed *in vitro*, by applying known concentrations of NPY in phosphate-buffered saline and recording the corresponding clamp current responses. These data are then used to generate a standard calibration curve, which is subsequently used to interpret real-time *in vivo* biosensor signals. Platinum wires were then inserted into the ventricular myocardium and 30 min were allowed for stabilization. Ag/AgCl disk ground pellets were inserted into intercostal muscle tissue. CI probes were cycled for 5 min to ensure functioning of the probes as well as to identify any basal NPY levels prior to stimulations, as previously described.^35,37^

NPY levels were measured using a custom-designed multichannel amplifier and IGOR Pro software (v. 7.08, WaveMetrics, Lake Oswego, OR, USA) interfaced with a HEKA LIH 8 + 8 system (HEKA Elektronik, Holliston, MA, USA) for command potential generation and data capture, as previously described.^35,37^ Data were filtered at a frequency of 1 kHz using a two-pole analogue Bessel filter, with digitization at 10 kHz. The electrodes were clamped at a specific command potential characterized by a step function waveform, varying from 0 mV to +100 mV for 20 ms, and then back to 0 mV, followed by a negative reset step to −5 mV. Capacitive currents required for the electrode's potential shift were analysed after subtracting specific capacitive currents from the overall data, thus isolating the current induced by NPY binding. NPY release was measured continuously, starting from 3 min prior to stimulations and for the entire 3-min duration of stimulation.

### 2.4 Statistical analyses

Data are reported as mean ± SEM. Global ventricular ARIs were calculated as the mean ARIs across all 56 electrodes. After confirmation of normality, two-tailed paired Student’s *t*-test was used to compare parameters between baseline and BSS, and baseline and BSS + VNS. One-way ANOVA was used to compare differences across conditions (BL, BSS, and BSS + VNS). A two-tailed paired Student’s *t*-test was also used to compare changes in parameters before and after BIIE or atropine infusions. For the rat stellate ganglia field stimulation studies, statistical analysis was performed using a two-tailed unpaired Student’s *t*-test when comparing NPY release in the CCH to CCH + atropine groups, also after confirmation of normality. A *P* ≤ 0.05 was considered statistically significant. All statistical analyses were performed with GraphPad Prism software (v9, Boston, MA, USA).

## 3. Results

### 3.1 MR subtype determination and effects of MR activation and blockade on NPY levels *ex vivo*

**Figure 1:**
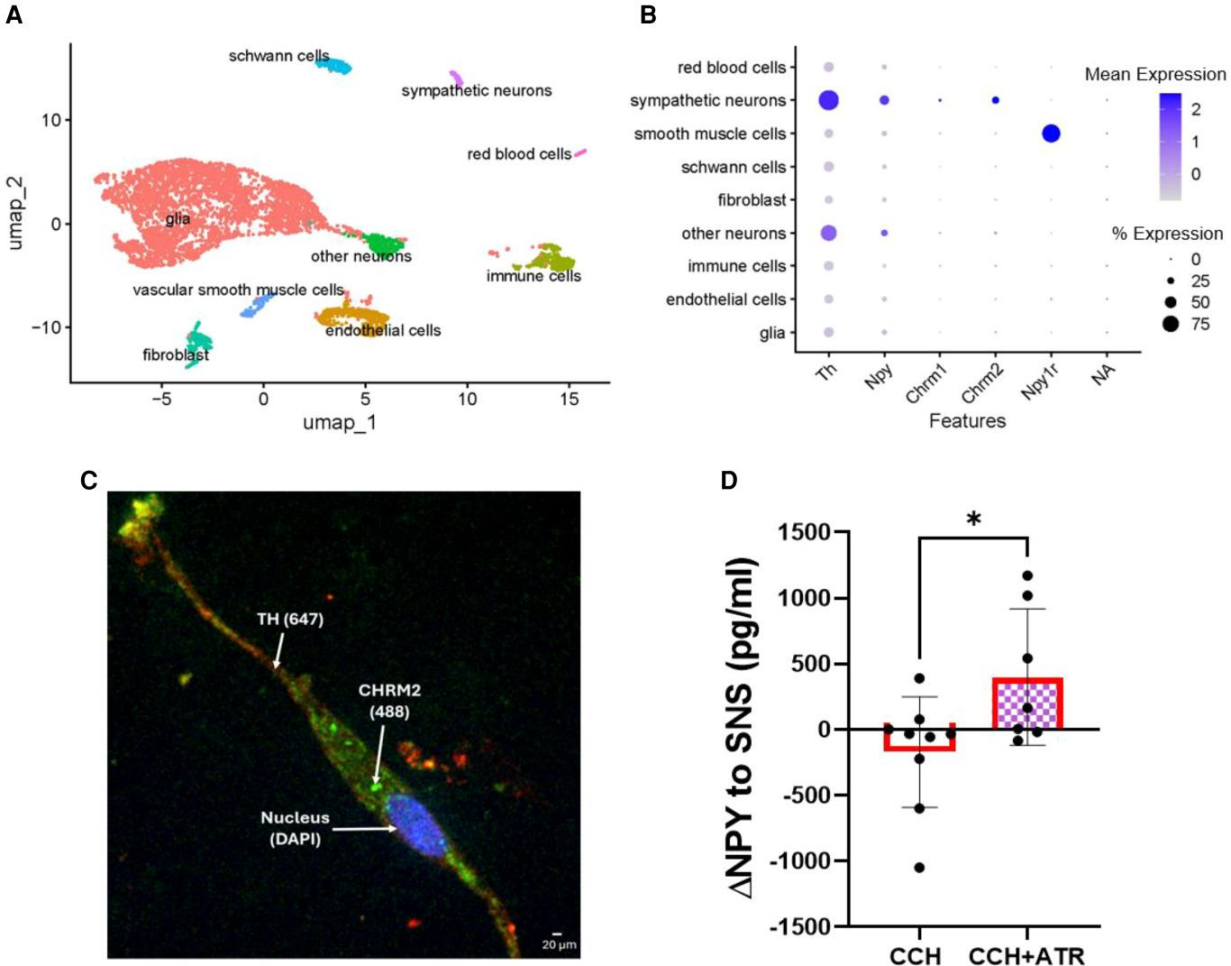
Identification and classification of sympathetic MRs and effect of cholinergic agonism on NPY release of rat stellate ganglia. (*A, B*) Single-cell RNA sequencing data from rat stellate ganglia was used to identify sympathetic neurons using expression of TH and NPY. Expression of MRs was assessed, and both TH and NPY-expressing neurons were found to also significantly express Chrm2 more than any other MR (Chrm1, Chrm3, and Chrm4). (*C*) Rat stellate ganglion neurons were cultured and Chrm2 expression confirmed in TH positive neurons. (*D*) Muscarinic agonist, CCH, reduced NPY release during isolated stellate ganglia field stimulations (SNS), and these effects were significantly mitigated by the addition of atropine (ATR). *P < 0.05, **P < 0.01, ***P < 0.001 for all experiments, *n* = 4 rats for scRNAseq; *n* = 18 rats (9 experiments) for CCH, *n* = 14 rats (7 experiments) for CCH + ATR. A two-tailed unpaired Student’s *t*-test was used to compare perfusate NPY levels in CCH vs. CCH + ATR rat stellate ganglia; scRNAseq data were analysed using Wilcoxon rank sum test to account for distribution variations and ultimately identify receptor subtypes. Note: smooth muscle cells refer to vascular smooth muscle cells.

Given prior studies demonstrating that VNS could suppress norepinephrine levels, we hypothesized that VNS could also potentially reduce NPY levels and aimed to determine whether these effects are mediated via cholinergic activation of pre-synaptic MRs on NPY-expressing sympathetic neurons. MR expression *ex vivo* in rat stellate ganglia was determined using scRNAseq and immunohistochemistry. As shown in *Figure 1A*, scRNAseq identified a distinct cluster of sympathetic neurons [with high expression of Cacna1b, Cav2.2; dopamine-β-hydroxylase; Dlg4, PSD-95; monoamine oxidase A; Slc6a2 noradrenaline transporter; tyrosine hydroxylase (TH); and NPY]. These neurons expressed predominantly the M2 subtype of MRs (Chrm2, *Figure 1B*), with very low transcript levels of Chrm1, 3 and 4, and no detectable Chrm5. Protein expression of Chrm2 in TH positive neurons cultured from the rat stellate ganglia was confirmed using immunohistochemistry (*Figure 1C*).

*Ex vivo*, high frequency field stimulation of rat stellate ganglia released NPY into the perfusate *in vitro*. The release of NPY was reduced after administration of the MR agonist, CCH. This effect was not observed when CCH with the addition of MR antagonist, atropine, was used (Δ-170 ± 140 pg/mL by CCH vs. Δ400 ± 196 pg/mL by CCH + atropine; *P* = 0.03, *Figure 1D*).

### 3.2 Effects of muscarinic blockade on NPY *in vivo*

**Figure 2:**
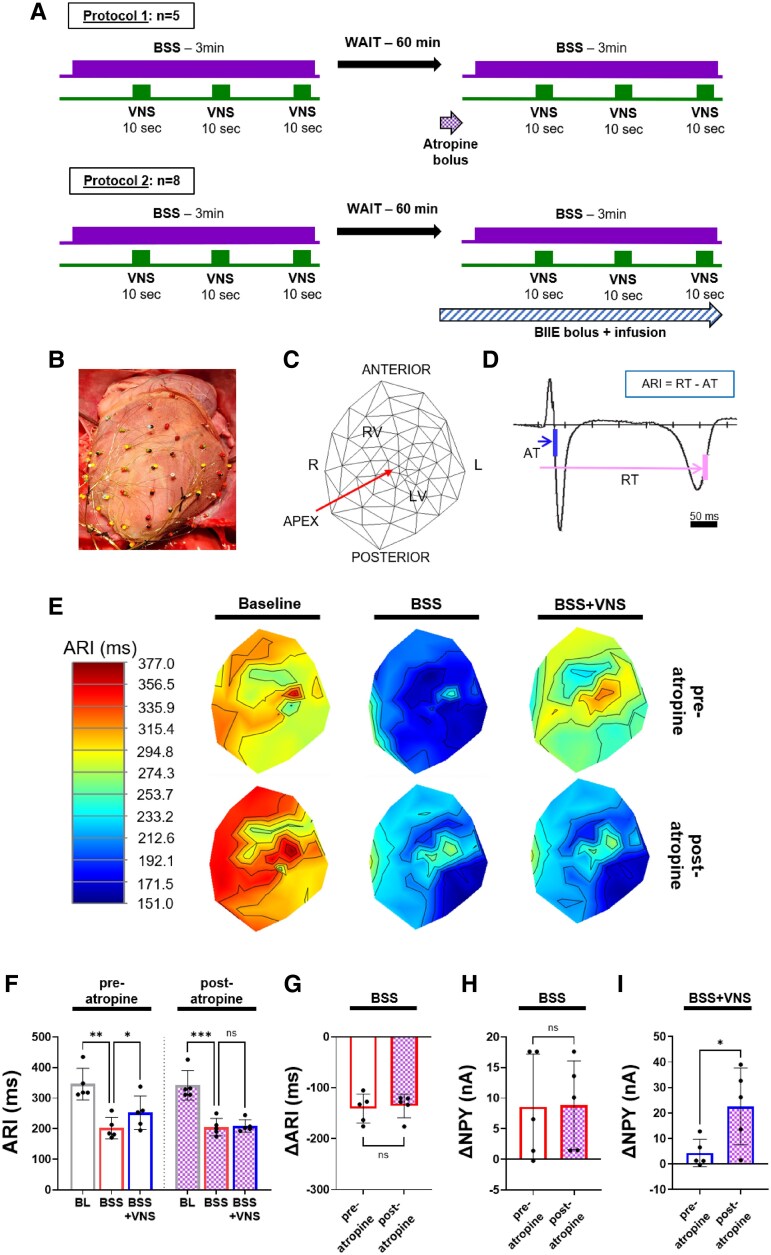
Effects of BSS with and without intermittent VNS on ventricular electrophysiological parameters and interstitial NPY levels before and after administration of atropine in intact pig model. (*A*) Study design and experimental protocol used to evaluate changes in the cardiac effects of BSS and BSS + VNS pre- and post-atropine (top) and Y2R blockade (bottom). (*B*) Schematic 2D-polar map representation of electrode locations on the 56-electrode sock placed on the ventricles. (*C*) Representative example of the sock electrode over the right and left ventricles *in vivo* used for measurement of unipolar electrograms. (*D*) Representative example of ARI calculations from a unipolar electrogram. ARIs were calculated as the difference between RT and AT of the unipolar electrogram. (*E*) Representative polar maps of ARIs at baseline (left), BSS (middle), and VNS during BSS (BSS + VNS, right), and respective changes after the administration of atropine are shown. (*F*) There is significant shortening in mean global ARIs pre- and post-atropine with BSS, while the effects of VNS during BSS on ARIs are blocked by atropine. (*G*) There is no difference in the peak ARI shortening during BSS pre- vs. post-atropine. (*H*) Notably, there is no significant change in NPY release (as measured by changes in NPY current from baseline) with BSS pre-atropine vs. post-atropine. (*I*) VNS reduces NPY levels during BSS, but this effect is no longer observed after atropine administration, suggesting that they were mediated via MRs. *P < 0.05, **P < 0.01, ***P < 0.001, *n* = 5 for all comparisons. One-way analysis of variance was used for comparisons of BL, BSS, and BSS + VNS parameters. A two-sided paired Student’s *t*-test was used for pre-atropine vs. post-atropine comparisons as well as BSS vs. BSS + VNS comparisons, *P* ≤ 0.05 was considered statistically significant.

To investigate if the *ex vivo* mechanisms underlying the reductions in NPY release with VNS were also present *in vivo*, haemodynamic and electrophysiological parameters and NPY profiles were measured in five pigs during continuous bilateral stellate ganglia stimulation with intermittent bilateral VNS, before and after atropine administration (*Figure 2A*). In line with prior studies, atropine mildly increased HR, left ventricular systolic pressure (LVSP), and inotropy (d*P*/d*t*_max_) by blocking basal vagal tone (see Supplementary material online, *Figure S1*).^25^ During BSS pre- and post-atropine, HR, LVSP, and d*P*/d*t*_max_ significantly increased from baseline (see Supplementary material online, *Figure S2*). Intermittent VNS reduced the haemodynamic and cardiac electrophysiological effects of BSS, and these effects were blocked by atropine (*Figure 2B–G* and Supplementary material online, *Figure S2*). The increase in NPY levels from pre-stimulation levels to peak levels with BSS post-atropine was similar to those observed with BSS pre-atropine (Δ8.6 ± 3.9 nA pre-atropine vs. Δ8.8 ± 3.2 nA post-atropine; *P* = 0.92, *Figure 2H*). Pre-atropine, VNS reduced NPY levels during BSS, however, after atropine administration, the reduction in interstitial NPY with VNS was significantly mitigated (NPY current pre-atropine with VNS: Δ4.3 ± 2.4 nA vs. NPY current post-atropine with VNS: Δ22.6 ± 15.1 nA, *P* = 0.04, *Figure 2I*), suggesting that the changes in myocardial NPY levels by VNS were mediated by MRs.

### 3.3 Haemodynamic and electrophysiological effects of BSS pre- vs. post-BIIE administration

**Figure 3:**
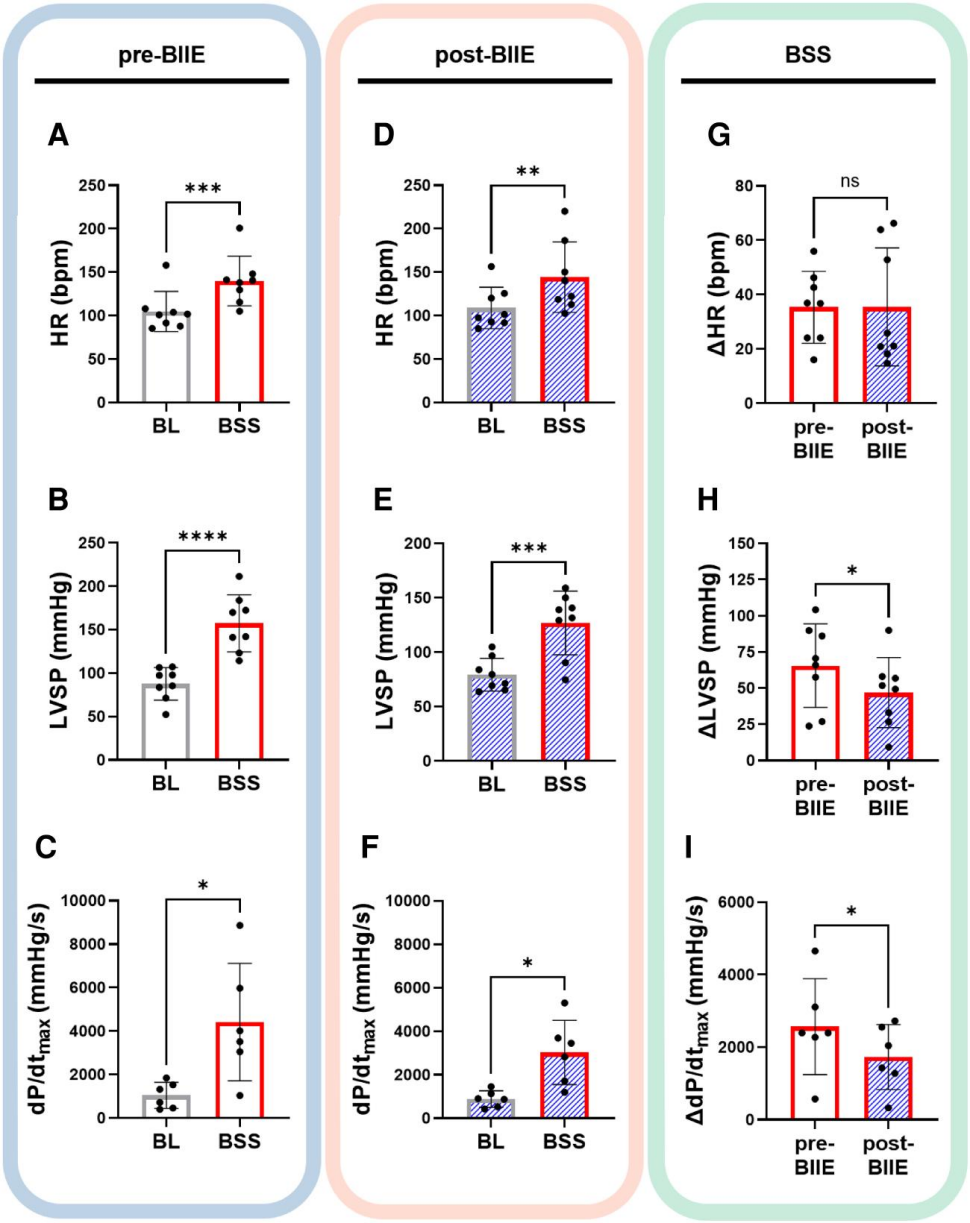
Cardiac effects of sympathetic stimulation before and after Y2R blockade. (*A*) BSS significantly increased mean HR, LVSP, and d*P*/d*t*_max_ from pre-stimulation, baseline (BL) values. (*D–F*) Significant increases in HR, LVSP, and d*P*/d*t*_max_ were also seen post-BIIE from baseline. (*G–I*) However, BIIE significantly decreased the effects of BSS on LVSP and d*P*/d*t*_max_, although significant changes in HR were not observed. *P < 0.05, **P < 0.01, ***P < 0.001, ****P < 0.0001, *n* = 8 for HR and LVSP, *n* = 6 for d*P*/d*t*_max_. A 2-sided paired Student’s *t*-test was used for comparisons of BL vs. BSS and pre-BIIE vs. post-BIIE parameters. *P* ≤ 0.05 was considered statistically significant.

Prior studies had suggested that *ex vivo*, NPY could reduce acetylcholine release by binding to Y2 receptors. These data suggested that NPY could significantly suppress vagal tone. To determine the influence of Y2R blockade on enhancing vagal tone *in vivo* (and potentially, therefore, inhibiting sympathetic tone), the effects of BSS pre- vs. post-BIIE, including on haemodynamic and electrophysiological parameters (during the first 45 s of sympathetic stimulation prior to VNS), were analysed (*Figure 3*). BSS significantly increased HR, LVSP, and inotropy (d*P*/d*t*_max_), both before and after administration of BIIE (*P* < 0.05, *Figure 3*). The effects of BSS on LVSP were significantly reduced post-BIIE (Δ65.6 ± 10.2 mmHg pre-BIIE vs. Δ46.9 ± 8.6 mmHg post-BIIE; *P* = 0.05, *Figure 3*). Increases in d*P*/d*t*_max_ were also significantly diminished after administration of BIIE (pre-BIIE d*P*/d*t*_max_ increased by Δ2566 ± 540.6 mmHg/s vs. Δ1725 ± 366.2 mmHg/s post-BIIE; *P* = 0.03, *Figure 3*). No change in peak HR was observed. Of note, infusion of Y2R blockade alone had no significant effect on haemodynamic parameters (see Supplementary material online, *Figure S3*).

**Figure 4:**
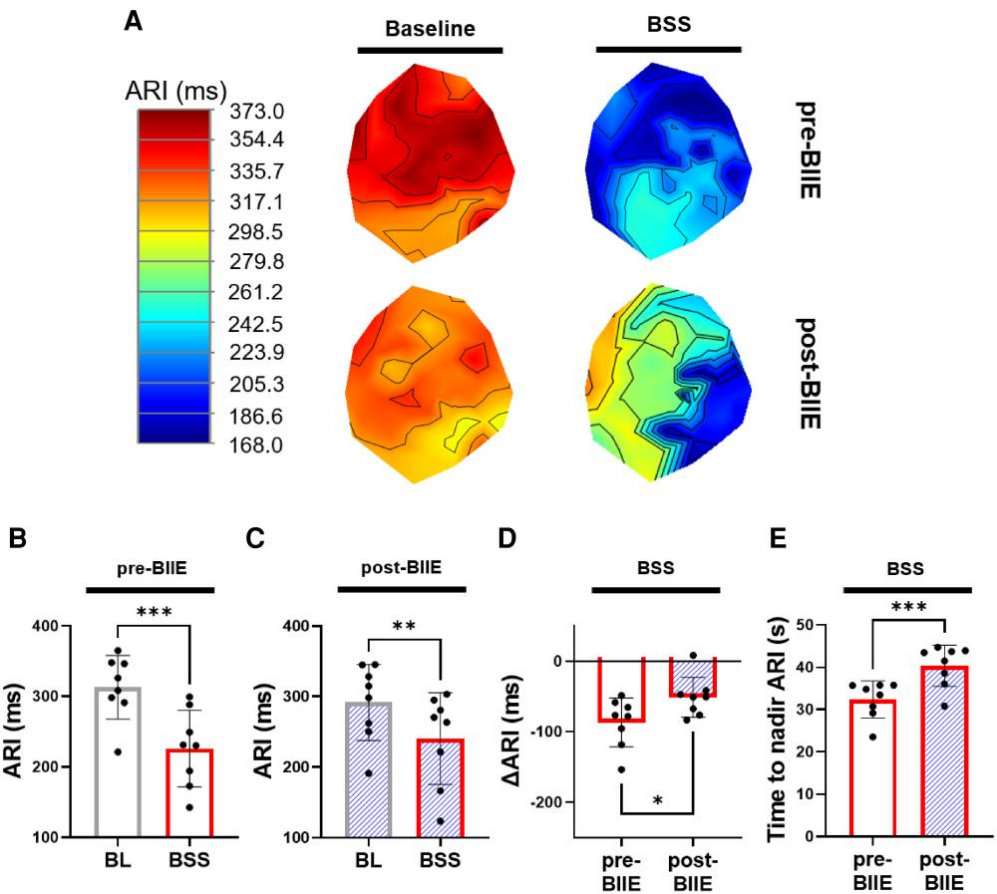
Effects of BSS on ventricular electrophysiological parameters before and after Y2R blockade. (*A*) Representative images of polar maps from one animal indicating baseline (left) and changes after BSS (right) before and after administration of the Y2R blocker, BIIE (prior to onset of VNS). (*B*, *C*) Mean global ARIs significantly shortened during BSS compared to pre-stimulation baseline values, both before and after BIIE infusion. (*D*) Effects of BSS on ARI shortening was significantly mitigated post-BIIE vs. pre-BIIE. (*E*) Time to nadir ARI shortening from the start of BSS was significantly longer post-BIIE vs. pre-BIIE, suggesting that a greater period of time was necessary for sympathetic stimulation to reach peak electrophysiological effects after the administration of the Y2R blocker. *P < 0.05, **P < 0.01, ***P < 0.001, *n* = 8 for all comparisons. A two-sided paired Student’s *t*-test used for comparisons of BL vs. BSS and pre-BIIE vs. post-BIIE parameters. *P* ≤ 0.05 was considered statistically significant. LV, left ventricle; RV, right ventricle; BSS, bilateral stellate ganglia stimulation.

To evaluate the influence of Y2R blockade on the electrophysiological effects of BSS, ARI recordings obtained during the first 45 s of BSS were analysed. ARIs significantly shortened with BSS both pre- and post-BIIE (*P* < 0.01, *Figure 4*). However, effects of BSS on ARIs post-BIIE were significantly less compared to pre-BIIE (Δ-87.1 ± 12.2 ms pre-BIIE vs. Δ-51.4 ± 10.0 ms post-BIIE; *P* = 0.02, *Figure 4D*). The time to achieve nadir ARI shortening with BSS was also prolonged post-BIIE compared to pre-BIIE (32.3 ± 1.5 s pre-BIIE vs. 40.3 ± 1.7 s post-BIIE; *P* = 0.0006, *Figure 4E*).

### 3.4 Haemodynamic and electrophysiological effects of VNS during BSS pre- vs. post-Y2R blockade

**Figure 5:**
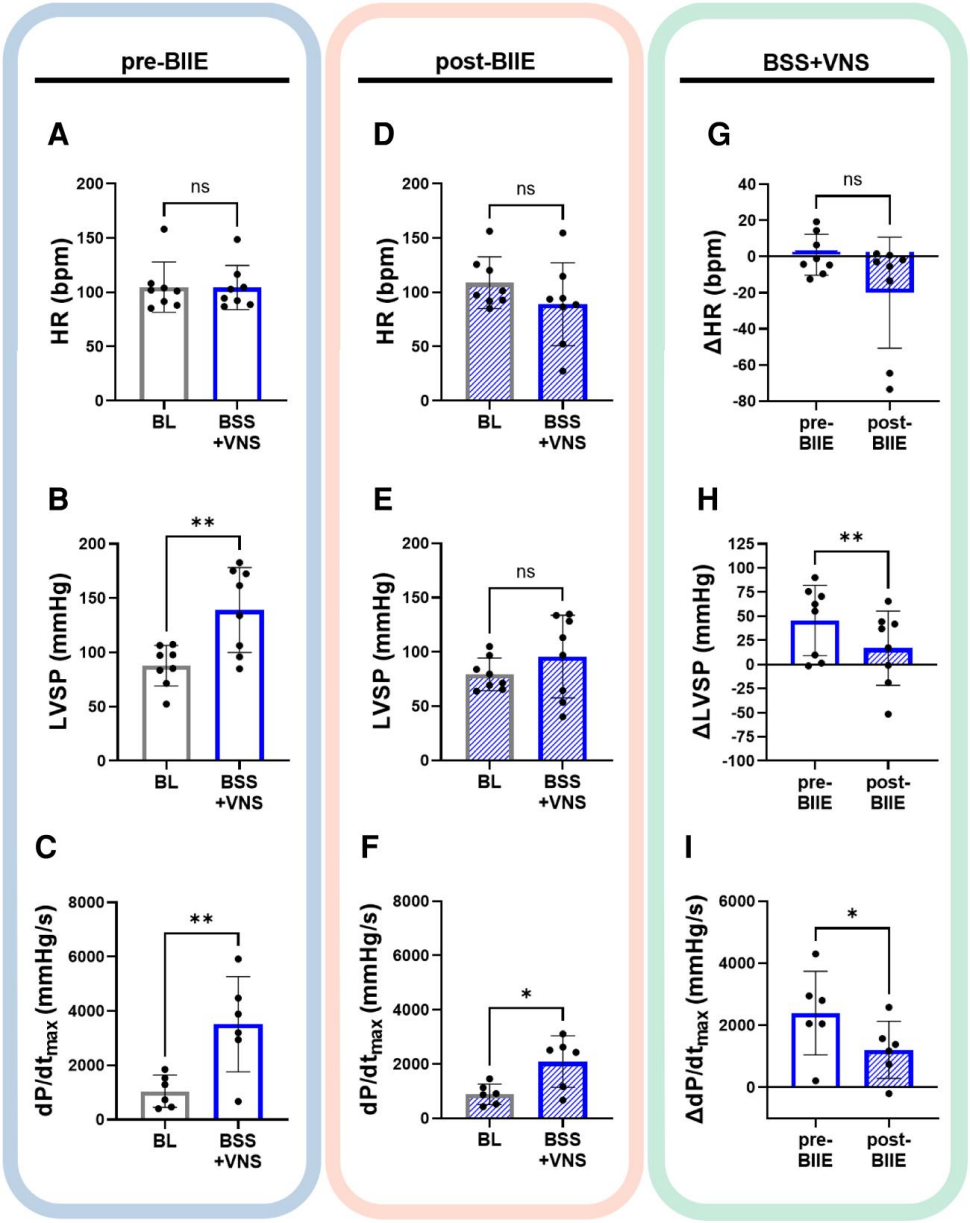
Haemodynamic effects of intermittent bilateral VNS during BSS. (*A–C*) Effects of intermittent VNS during BSS on HR, LVSP, and d*P*/d*t*_max_ pre-BIIE are shown and compared to pre-stimulation (baseline) values (*D–F*). Effects of intermittent VNS on HR, LVSP, and d*P*/d*t*_max_ during BSS are shown post-BIIE. (*E–G*) The effects of VNS during BSS on LVSP and d*P*/d*t*_max_ were significantly enhanced by Y2R blockade, bringing these parameters to near baseline (pre-BSS) values, despite ongoing sympathetic stimulations; effects on HR remained unchanged. *P < 0.05, **P < 0.01, ***P < 0.001, *n* = 8 for HR and LVSP, *n* = 6 for d*P*/d*t*_max_. Comparisons between BL and BSS + VNS as well as pre- vs. post-BIIE parameters were performed using a two-sided paired Student’s *t*-test. *P* ≤ 0.05 was considered statistically significant. BSS, bilateral stellate ganglia stimulation; BSS + VNS, effects of VNS during bilateral stellate ganglia stimulation.

Consistent with the results observed in the pigs that underwent VNS prior to atropine administration, VNS during BSS moderately reduced the haemodynamic effects of sympathetic stimulation on HR and LVSP (*Figure 5*). After BIIE administration, the effects of VNS during BSS on HR were unchanged (*Figure 5G*). However, the effects of VNS during BSS on LVSP were significantly greater post-BIIE vs. pre-BIIE (*Figure 5H*), with VNS during sympathetic stimulation returning LV pressures back to near baseline values. Similarly, VNS during BSS reduced d*P*/d*t*_max_, with more significant reductions observed post-BIIE vs. pre-BIIE (*Figure 5I*), suggesting a strengthening of the effects of VNS post-Y2R blockade.

**Figure 6:**
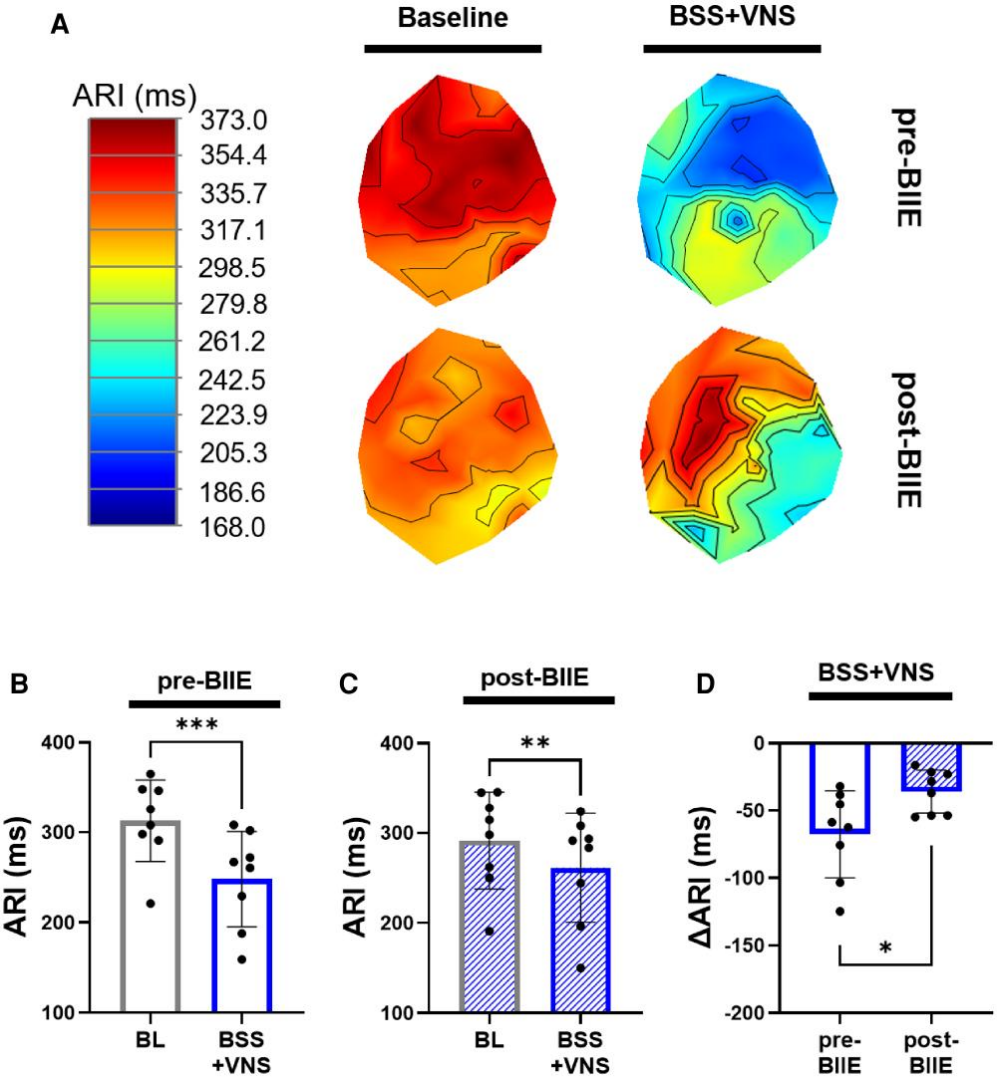
Effects of VNS during BSS on ventricular electrophysiology before and after Y2R blockade. (*A*) Representative polar map images indicating baseline (left) and changes during intermittent VNS with BSS (right) before and after administering BIIE. (*B*) VNS during BSS pre-BIIE mitigated sympathetic effects on ARI shortening compared to baseline. (*C*) After BIIE administration, mitigation of ARI shortening was still observed during intermittent VNS. (*D*) However, a significant increase in vagally induced ARI effects was observed post-BIIE vs. pre-BIIE. *P < 0.05, **P < 0.01, ***P < 0.001, *n* = 8 for all comparisons. A two-sided paired Student’s *t*-test was used for BL vs. BSS + VNS and pre-BIIE vs. post-BIIE comparisons, *P* ≤ 0.05 was considered statistically significant. BSS, bilateral stellate ganglia stimulation; BSS + VNS, effects of VNS during bilateral stellate ganglia stimulation.

Effects of BSS on ARI shortening were mitigated with the onset of VNS both pre- and post-BIIE (*P* < 0.01, *Figure 6A–C*). However, post-BIIE, the electrophysiological effects of VNS were further enhanced (*Figure 6D*), with the return of ARI shortening closer to baseline values during sympathetic stimulation—indicating an enhanced effect of VNS mediated attenuation of ARI shortening following Y2R blockade.

### 3.5 Effects of VNS on NPY release during BSS

**Figure 7:**
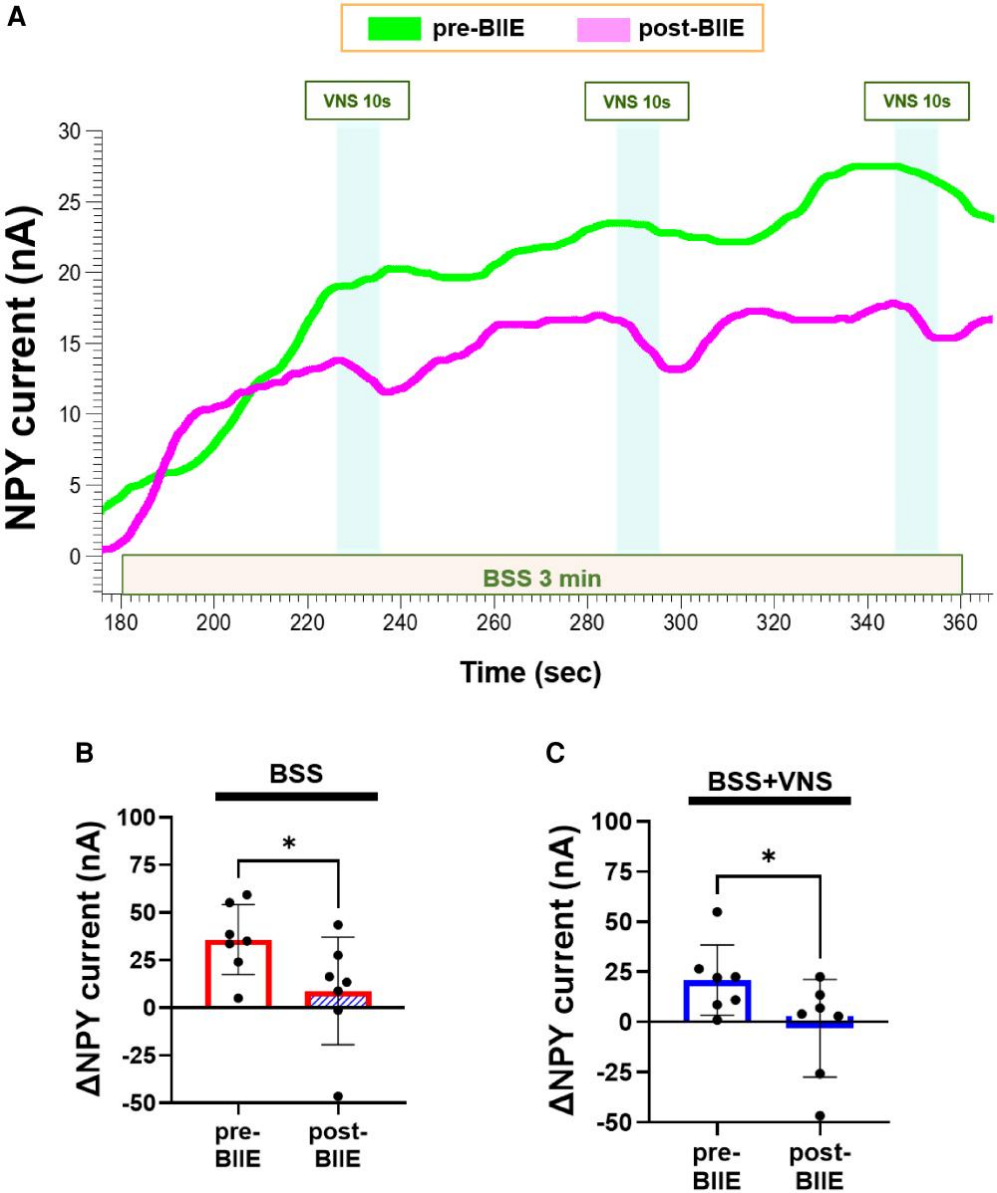
*In vivo* measurements of real-time ventricular interstitial levels using CI. (*A*) Representative example of *in vivo* myocardial NPY release profiles during sympathoexcitation and reduction of myocardial interstitial NPY by intermittent VNS during BSS. Post-BIIE (purple tracing), VNS effects on NPY levels were greater compared to pre-BIIE (green tracing), despite overall NPY levels being reduced by BIIE. (*B*) Post-BIIE, quantified NPY release profiles across animals during sympathetic stimulation demonstrated significantly less NPY release compared to pre-BIIE. (*C*) Pre-BIIE, VNS stimulation reduced the release of NPY. The effect of VNS during sympathetic stimulation on NPY current/release post-BIIE was even greater, resulting in minimal to no NPY release. *P < 0.05, **P < 0.01, *n* = 7 for all comparisons. A two-sided paired Student’s *t*-test was used for BL vs. BSS and pre-BIIE vs. post-BIIE comparisons, *P* ≤ 0.05 was considered statistically significant. BSS, bilateral stellate ganglia stimulation; BSS + VNS, effects of VNS during bilateral stellate ganglia stimulation.

As in the atropine studies, BSS led to the release of NPY pre- and post-BIIE, though NPY release was significantly less post-BIIE (Δ35.9 ± 7 nA from baseline pre-BIIE vs. Δ8.8 ± 10.7 nA post-BIIE; *P* = 0.03, *Figure 7A* and *B*). In line with the *ex vivo* studies of carbamylcholine, intermittent VNS significantly reduced NPY levels during sympathetic activation, an effect that was present both pre- and post-BIIE. However, the reduction in NPY levels during VNS were significantly enhanced after Y2R blockade (*Figure 7C*).

## 4. Discussion

### 4.1 Major findings

In this study, single-cell RNA sequencing and immunohistochemistry were used to demonstrate the presence of M2 receptors as the predominant MR subtype on NPY-expressing sympathetic neurons in the stellate ganglia. A reduction in NPY levels was observed with muscarinic activation in field sympathetic stimulation studies *ex vivo* and with VNS *in vivo*, effects that were blocked by the MR blocker, atropine. Y2R blockade *in vivo* in the porcine model significantly enhanced the effects VNS on inotropy, LVSP, and action potential duration, but not HR. In addition, Y2R blockade reduced the effects of BSS on systolic blood pressure and inotropy. These data provide important insights on the sympathovagal crosstalk involving NPY, demonstrating that the reduction in NPY release by VNS is mediated via MRs and that Y2R blockade may be potentially used to enhance vagal and reduce sympathetic ventricular effects, without causing significant bradycardia.

### 4.2 Effects of VNS on interstitial myocardial NPY levels

Given the presence of M2 receptors on NPY-expressing sympathetic neurons isolated from the rat stellate ganglia, the effects of carbamylcholine and atropine on NPY release were evaluated *ex vivo* to determine if muscarinic activation could reduce NPY levels and if these effects were blocked by muscarinic blockade. These studies were then used to inform the *in vivo* studies in pigs. To our knowledge, this is the first study to demonstrate that VNS can reduce interstitial myocardial NPY levels, and that the mechanism for this action is mediated via M2 receptors. Levy and colleagues had previously shown that high levels of vagal stimulation can reduce coronary sinus norepinephrine levels.^15^ It has also been previously reported that VNS can mitigate the haemodynamic and electrophysiological effects of sympathetic activation *in vivo* in infarcted porcine hearts.^30^ These cardiac effects of VNS were previously thought to be due (i) inhibition of norepinephrine release^15,38,39^ and (ii) due to myocardial MR activation by acetylcholine, which can, via activation of inhibitory G proteins, oppose the effects of norepinephrine on adrenergic-receptor mediated cAMP signalling.^6,40–42^ Our findings take these studies a step further by showing that VNS can also suppress interstitial ventricular NPY release via a pre-synaptic sympathetic MR type 2 mechanism. Yang *et al.*^43^ had suggested that M2 receptor stimulation can inhibit calcium currents in rat sympathetic neurons *in vitro*, and our scRNAseq further demonstrates that stellate ganglia NPY-expressing sympathetic neurons highly express the M2R subtype, which could underlie these observations.

### 4.3 Y2 receptor blockade attenuates cardiac effects of sympathoexcitation

Myocardial injury and infarction cause sympathetic activation, leading to the release of not only norepinephrine but also the sympathetic co-transmitter NPY. Additionally, NPY levels are elevated in the setting of heart failure and higher levels portend a poorer prognosis, including increased mortality.^44–47^ NPY can interact with Y1 receptors which exist on both pre-synaptic sympathetic nerve terminals and on cardiomyocytes. Binding of NPY to Y1 receptors has been shown to have direct electrophysiological effects on action potential duration and arrhythmogenesis, independent of norepinephrine.^12,13,23^ Prior studies did not show a significant effect of Y1 receptor blockade on NPY release beyond β-blocker therapy in pig ventricles;^12^ however, in both *ex vivo* and *in vivo* models, Y1 receptor blockade in addition to high dose β-blockers was required to fully blunt the ventricular electrophysiological effects of sympathetic stimulation.^12,13^ Finally, Y1 receptors have been shown to be present on blood vessels, including coronary arteries, and their activation by NPY causes significant coronary and systemic vasoconstriction. In the setting of acute myocardial infarction or other conditions of heightened sympathetic tone, NPY release can, therefore, further exacerbate ischaemia.^48,49^ In the current study, it’s possible that the reduced effects of sympathetic nerve stimulation on systolic pressures after Y2R blockade were mediated in part by the reduction in NPY release, leading to decreased vasoconstriction.^17^ These findings are of particular electrophysiological significance given that, unlike norepinephrine, which has a short half-life (less than 30 s) and a 90% clearance time of approximately 42 s,^18^ NPY has a much longer half-life (∼20 min) and a 90% clearance time of more than 35 min.^19^ Consequently, NPY can suppress vagal tone and mediate its pro-arrhythmic electrophysiological effects for more extended periods.^18^ Based on these prior findings, we aimed to determine if mitigating the effects of NPY on acetylcholine release via Y2R blockade *in vivo* may enhance vagal tone so that the haemodynamic and electrophysiological effects of sympathetic stimulation may be mitigated. In comparing the haemodynamic parameters during sympathetic stimulation before and after the administration of BIIE, we observed a significant reduction in the effects of BSS on LVSP and d*P*/d*t*_max_ after Y2R blockade. In addition, administering BIIE significantly reduced the effects of sympathetic stimulation on ventricular ARI shortening, and the time to reach nadir ARIs with BSS was also prolonged.

It's important to note that while Y2 receptors are predominantly found on parasympathetic nerve endings in the heart and colocalize with choline acetyl transferase expressing neurons in sinoatrial tissue,^23^ they have also been reported to be present on cardiomyocytes,^50^ and their activation on rat myocytes was associated with negative inotropic effects *in vitro*.^50,51^ In this study, while BIIE enhanced vagal effects and mitigated the effects of sympathetic stimulation, it did not significantly alter basal haemodynamic parameters.

### 4.4 Y2 receptor blockade augments the effects of VNS

VNS has been shown to mitigate the cardiac effects of sympathetic activation and to be anti-arrhythmic in multiple pre-clinical studies.^27,30,52,53^ Yet several clinical trials of VNS in heart failure have failed to show clinical benefit, an outcome that has been attributed, in part, to the stimulation parameters used^54,55^ and the fact that electrical stimulation of the vagus nerve in humans often has off-target effects (including laryngeal nerve stimulation resulting in cough or hoarseness), reducing the ability to reach stimulation currents needed to achieve therapeutic efficacy.^54–57^ Hence, augmenting the end-organ effects of VNS through a pharmacological method could prove potentially beneficial.

In this study, we tested the hypothesis that NPY Y2R blockade could enhance the effects of VNS, without the need for increasing stimulation parameters. Using a porcine model, we demonstrate that the cardiac effects of VNS on ventricular electrophysiological parameters, inotropy, and ventricular systolic pressure were amplified after Y2R blockade. Interestingly, no significant effect on HR was observed. Notably, Potter and colleagues had reported that *in vivo* NPY infusion reduces the effects of vagal stimulation on HR in a canine model.^19^ Herring *et al.* and Schwertferger *et al.* further built on this finding by showing that in *ex vivo* human atrial appendage tissue and guinea pigs sinoatrial tissue, NPY acts via pre-synaptic Y2 receptors on parasympathetic nerve terminals to reduce acetylcholine.^6,17^ The fact that Y2R blockade *in vivo* failed to enhance the effects of VNS on HR may be due to several factors. Sympathetic stimulation has been shown to lead to the release of other co-transmitters beyond NPY, such as galanin, which can also reduce acetylcholine release, and may mask the enhancement of bradycardia by Y2R.^23^ In the sinoatrial tissue experiments above, 10 nM of NPY did not alter HR responses to vagal nerve stimulation and 100 and 250 nM of NPY were needed to observe decreases on VNS effects on HR.^6^ Given that vagal innervation of the sinoatrial node, unlike the ventricular myocardium, is extensive,^14,58^ it is also possible that the levels of NPY released during sympathoexcitation *in vivo* in the current study were not sufficient to reduce vagal effects on HR. In contrast, the ventricles, which have sparser parasympathetic innervation,^14,58^ may be more sensitive to the enhanced effects vagal activation. These findings also highlight the importance of *in vivo* studies in evaluating the effects of receptor agonists and antagonists. Our data suggest that Y2R blockers may be used not only to reduce the electrophysiological effects of sympathoexcitation but also as a potential adjuvant therapy to enhance the ventricular electrophysiological effects of VNS, potentially allowing for better efficacy and titration of VNS parameters.

### 4.5 Limitations

It's important to note that acetylcholine release can also be associated with the release of other co-transmitters, such as vasoactive intestinal peptide (VIP). VIP has been shown to be a potent vasodilator and is released with high levels of VNS.^59,60^ VIP release requires high frequencies of vagal stimulation (>20 Hz) and is also reportedly associated with atrial fibrillation.^28,57,59^ To avoid atrial arrhythmias, our VNS stimulation was limited to 10 Hz, which is also in line with prior studies showing beneficial ventricular effects.^28,57^ Hence, any VIP-related effects on our findings are likely to be minimal. Several studies have shown that adrenergic receptors on sympathetic nerve terminals can impact the release of norepinephrine and NPY. Pre-synaptic α2-adrenergic receptors are known to mediate rapid, phasic inhibition norepinephrine, and NPY release via Gi-coupled signalling pathways, acting as a short-term negative feedback mechanism on sympathetic tone.^61,62^ Pharmacologic studies have shown that α2R antagonism with yohimbine increases both norepinephrine and NPY release, consistent with this role. By contrast, β-adrenergic receptors on pre-synaptic sympathetic nerve terminals facilitate NPY release, and it has been previously demonstrated that propranolol reduces NPY release from sympathetic nerve endings during sympathetic nerve stimulation.^37^ Given this prior data on the pre-synaptic adrenergic mechanisms that govern norepinephrine and NPY release, our current study focused on sympathovagal crosstalk via Y2R and MRs. It’s possible, however, that the reduced effects of sympathetic stimulation and greater effects of vagal stimulation on haemodynamic and electrophysiological parameters after Y2R blockade may be, at least in part, altered via adrenergic activation of α2 and β-adrenergic receptors due to reduced norepinephrine release. Given that these receptors appear to have opposing effects on NPY release, evaluation of the cardiac effects of Y2R in combination with β- and/or α2-blockers requires carefully designed studies in the future.

Isoflurane has been shown to reduce responses to autonomic stimulations. To offset these effects, anaesthesia was switched to alpha-chloralose after surgical procedures were completed. Amongst agents used for general anaesthesia, α-chloralose has been reported to interfere with autonomic reflexes the least,^63–67^ and therefore, all *in vivo* recordings were obtained with this agent. Despite this, we cannot fully exclude the possibility of autonomic effects of alpha-chloralose. Therefore, all measurements (pre- and post-interventions) were obtained after animals had been stabilized on alpha-chloralose and changes in parameters from baseline (in addition to absolute values) were evaluated to account for any significant effects that may be present due to the duration/length of alpha-chloralose administration. ARI effects were not corrected for HR, though Y2R blockade still reduced effects of sympathetic stimulation on ARIs, despite not causing a significant change in HR. The current study evaluated the acute effects of VNS pre- and post-Y2R blocker; therefore, the chronic effects of Y2R blockade remain to be investigated. Also, the current study used rat stellate ganglia to evaluate for the presence and subtypes of MR on NPY-expressing neurons; future experiments are needed to directly characterize this in porcine tissue. Finally, although suggestive, definitive conclusions regarding the anti-arrhythmic effects of Y2R blockers cannot drawn from these studies, as inducibility testing was not performed in these healthy animals, given that in our experience, ventricular arrhythmia inducibility is low in pigs with structurally normal hearts.

Translational perspective This study demonstrates that vagal nerve stimulation has direct effects on NPY release via a pre-synaptic sympathetic MR type 2 mechanism. It also highlights the potential therapeutic benefit of targeting neuropeptide Y2 receptors to modulate autonomic tone *in vivo*. Our findings demonstrate that Y2 receptor blockade can mitigate the effects of sympathoexcitation and enhance the effects of vagal nerve stimulation. These data suggest that a combined bioelectric and pharmacological approach could be beneficial in restoring autonomic balance in the setting of cardiovascular diseases where sympathoexcitation and vagal dysfunction persist.

## 5. Conclusions

Cholinergic activation with VNS or muscarinic agonists reduces NPY levels, and these effects are likely mediated via MR subtype 2, a novel finding of this study. Furthermore, Y2R blockade can mitigate both the haemodynamic and electrophysiological effects of sympathoexcitation and enhance the cardiac effects of VNS on inotropy, LVSP, and action potential duration during sympathetic activation. These data suggest that targeting Y2R can potentially improve sympathovagal balance and serve as a potential neuromodulatory target. These results also provide important insights into the sympathovagal crosstalk involving NPY, which has been shown to have pro-arrhythmic effects in patients after myocardial infarction.

## Supplementary Material

cvaf180_Supplementary_Data

## Data Availability

*Rat studies:* The RNA sequencing data from the rat stellate ganglion are available in the Gene Expression Omnibus repository, accession number GSE144027. These data include scRNAseq results identifying the expression of MR subtypes in the stellate ganglion. *Porcine studies*: The supporting data for this study are available from the corresponding author upon reasonable request. This includes haemodynamic and electrophysiological measurements.

## References

[1] Wu P, Vaseghi M. The autonomic nervous system and ventricular arrhythmias in myocardial infarction and heart failure. Pacing Clin Electrophysiol 2020;43:172–180. 31823401 10.1111/pace.13856 PMC7723010

[2] van Weperen VYH, Vaseghi M. Cardiac vagal afferent neurotransmission in health and disease: review and knowledge gaps. Front Neurosci 2023;17:1192188. 37351426 10.3389/fnins.2023.1192188 PMC10282187

[3] Florea VG, Cohn JN. The autonomic nervous system and heart failure. Circ Res 2014;114:1815–1826. 24855204 10.1161/CIRCRESAHA.114.302589

[4] Dusi V, De Ferrari GM, Schwartz PJ. There are 100 ways by which the sympathetic nervous system can trigger life-threatening arrhythmias. Eur Heart J 2020;41:2180–2182. 31985796 10.1093/eurheartj/ehz950

[5] van Weperen VYH, Ripplinger CM, Vaseghi M. Autonomic control of ventricular function in health and disease: current state of the art. Clin Auton Res 2023;33:491–517. 37166736 10.1007/s10286-023-00948-8 PMC10173946

[6] Herring N, Lokale MN, Danson EJ, Heaton DA, Paterson DJ. Neuropeptide Y reduces acetylcholine release and vagal bradycardia via a Y2 receptor-mediated, protein kinase C-dependent pathway. J Mol Cell Cardiol 2008;44:477–485. 17996892 10.1016/j.yjmcc.2007.10.001

[7] Lundberg JM, Terenius L, Hökfelt T, Martling CR, Tatemoto K, Mutt V, Polak J, Bloom S, Goldstein M. Neuropeptide Y (NPY)-like immunoreactivity in peripheral noradrenergic neurons and effects of NPY on sympathetic function. Acta Physiol Scand 1982;116:477–480. 6763452 10.1111/j.1748-1716.1982.tb07171.x

[8] Potter EK . Neuropeptide Y as an autonomic neurotransmitter. Pharmacol Ther 1988;37:251–273. 2898787 10.1016/0163-7258(88)90028-9

[9] Huang W, Zhang Q, Qi H, Shi P, Song C, Liu Y, Sun H. Deletion of neuropeptide Y attenuates cardiac dysfunction and apoptosis during acute myocardial infarction. Front Pharmacol 2019;10:1268. 31708788 10.3389/fphar.2019.01268 PMC6821782

[10] Gibbs T, Tapoulal N, Shanmuganathan M, Burrage MK, Borlotti A, Banning AP, Choudhury RP, Neubauer S, Kharbanda RK, Ferreira VM, Channon KM, Herring N; OxAMI (Oxford Acute Myocardial Infarction) Study. Neuropeptide-Y levels in ST-segment-elevation myocardial infarction: relationship with coronary microvascular function, heart failure, and mortality. J Am Heart Assoc 2022;11:e024850. 35766271 10.1161/JAHA.121.024850 PMC9333365

[11] McDowell K, Adamson C, Jackson C, Campbell R, Welsh P, Petrie MC, McMurray JJV, Jhund PS, Herring N. Neuropeptide Y is elevated in heart failure and is an independent predictor of outcomes. Eur J Heart Fail 2024;26:107–116. 37937329 10.1002/ejhf.3085

[12] Hoang JD, Salavatian S, Yamaguchi N, Swid MA, David H, Vaseghi M. Cardiac sympathetic activation circumvents high-dose beta blocker therapy in part through release of neuropeptide Y. JCI Insight 2020;5:e135519. 32493842 10.1172/jci.insight.135519 PMC7308065

[13] Kalla M, Hao G, Tapoulal N, Tomek J, Liu K, Woodward L, Dall'Armellina E, Banning AP, Choudhury RP, Neubauer S, Kharbanda RK, Channon KM, Ajijola OA, Shivkumar K, Paterson DJ, Herring N. The cardiac sympathetic co-transmitter neuropeptide Y is pro-arrhythmic following ST-elevation myocardial infarction despite beta-blockade. Eur Heart J 2020;41:2168–2179. 31834357 10.1093/eurheartj/ehz852 PMC7299634

[14] Levy M, Zieske H. Comparison of the cardiac effects of vagus nerve stimulation and of acetylcholine infusions. Am J Physiol 1969;216:890–897. 5775885 10.1152/ajplegacy.1969.216.4.890

[15] Levy MN, Blattberg B. Effect of vagal stimulation on the overflow of norepinephrine into the coronary sinus during cardiac sympathetic nerve stimulation in the dog. Circ Res 1976;38:81–84. 1245024 10.1161/01.res.38.2.81

[16] Yamaguchi N, Yamakawa K, Rajendran PS, Takamiya T, Vaseghi M. Antiarrhythmic effects of vagal nerve stimulation after cardiac sympathetic denervation in the setting of chronic myocardial infarction. Heart Rhythm 2018;15:1214–1222. 29530832 10.1016/j.hrthm.2018.03.012 PMC6245660

[17] Schwertfeger E, Klein T, Vonend O, Oberhauser V, Stegbauer J, Rump LC. Neuropeptide Y inhibits acetylcholine release in human heart atrium by activation of Y2-receptors. Naunyn Schmiedebergs Arch Pharmacol 2004;369:455–461. 15103451 10.1007/s00210-004-0930-9

[18] Warner MR, Senanayake PD, Ferrario CM, Levy MN. Sympathetic stimulation-evoked overflow of norepinephrine and neuropeptide Y from the heart. Circ Res 1991;69:455–465. 1776971 10.1161/01.res.69.2.455

[19] Potter EK . Cardiac vagal action and plasma levels of neuropeptide Y following intravenous injection in the dog. Neurosci Lett 1987;77:243–247. 3601235 10.1016/0304-3940(87)90594-5

[20] Davis H, Herring N, Paterson DJ. Downregulation of M current is coupled to membrane excitability in sympathetic neurons before the onset of hypertension. Hypertension 2020;76:1915–1923. 33040619 10.1161/HYPERTENSIONAHA.120.15922 PMC8360673

[21] Kupari J, Häring M, Agirre E, Castelo-Branco G, Ernfors P. An atlas of vagal sensory neurons and their molecular specialization. Cell Rep 2019;27:2508–2523.e4. 31116992 10.1016/j.celrep.2019.04.096 PMC6533201

[22] Lu CJ, Hao G, Nikiforova N, Larsen HE, Liu K, Crabtree MJ, Li D, Herring N, Paterson DJ. CAPON modulates neuronal calcium handling and cardiac sympathetic neurotransmission during dysautonomia in hypertension. Hypertension 2015;65:1288–1297. 25916729 10.1161/HYPERTENSIONAHA.115.05290 PMC4487208

[23] Herring N, Cranley J, Lokale MN, Li D, Shanks J, Alston EN, Girard BM, Carter E, Parsons RL, Habecker BA, Paterson DJ. The cardiac sympathetic co-transmitter galanin reduces acetylcholine release and vagal bradycardia: implications for neural control of cardiac excitability. J Mol Cell Cardiol 2012;52:667–676. 22172449 10.1016/j.yjmcc.2011.11.016 PMC3314977

[24] Kalla M, Chotalia M, Coughlan C, Hao G, Crabtree MJ, Tomek J, Bub G, Paterson DJ, Herring N. Protection against ventricular fibrillation via cholinergic receptor stimulation and the generation of nitric oxide. J Physiol 2016;594:3981–3992. 26752781 10.1113/JP271588 PMC4794549

[25] Yamakawa K, Rajendran PS, Takamiya T, Yagishita D, So EL, Mahajan A, Shivkumar K, Vaseghi M. Vagal nerve stimulation activates vagal afferent fibers that reduce cardiac efferent parasympathetic effects. Am J Physiol Heart Circ Physiol 2015;309:H1579–H1590. 26371172 10.1152/ajpheart.00558.2015 PMC4666973

[26] Yamakawa K, So EL, Rajendran PS, Hoang JD, Makkar N, Mahajan A, Shivkumar K, Vaseghi M. Electrophysiological effects of right and left vagal nerve stimulation on the ventricular myocardium. Am J Physiol Heart Circ Physiol 2014;307:H722–H731. 25015962 10.1152/ajpheart.00279.2014 PMC4187397

[27] Vaseghi M, Salavatian S, Rajendran PS, Yagishita D, Woodward WR, Hamon D, Yamakawa K, Irie T, Habecker BA, Shivkumar K. Parasympathetic dysfunction and antiarrhythmic effect of vagal nerve stimulation following myocardial infarction. JCI Insight 2017;2:e86715. 28814663 10.1172/jci.insight.86715 PMC5621871

[28] Ardell JL, Nier H, Hammer M, Southerland EM, Ardell CL, Beaumont E, KenKnight BH, Armour JA. Defining the neural fulcrum for chronic vagus nerve stimulation: implications for integrated cardiac control. J Physiol 2017;595:6887–6903. 28862330 10.1113/JP274678 PMC5685838

[29] Oliveira M, da Silva MN, Geraldes V, Xavier R, Laranjo S, Silva V, Postolache G, Ferreira R, Rocha I. Acute vagal modulation of electrophysiology of the atrial and pulmonary veins increases vulnerability to atrial fibrillation. Exp Physiol 2011;96:125–133. 20952490 10.1113/expphysiol.2010.053280

[30] Hoang JD, Yamakawa K, Rajendran PS, Chan CA, Yagishita D, Nakamura K, Lux RL, Vaseghi M. Proarrhythmic effects of sympathetic activation are mitigated by vagal nerve stimulation in infarcted hearts. JACC Clin Electrophysiol 2022;8:513–525. 35450607 10.1016/j.jacep.2022.01.018 PMC9034056

[31] Yagishita D, Chui RW, Yamakawa K, Rajendran PS, Ajijola OA, Nakamura K, So EL, Mahajan A, Shivkumar K, Vaseghi M. Sympathetic nerve stimulation, not circulating norepinephrine, modulates T-peak to T-end interval by increasing global dispersion of repolarization. Circ Arrhythm Electrophysiol 2015;8:174–185. 25532528 10.1161/CIRCEP.114.002195 PMC4405126

[32] Irie T, Yamakawa K, Hamon D, Nakamura K, Shivkumar K, Vaseghi M. Cardiac sympathetic innervation via middle cervical and stellate ganglia and antiarrhythmic mechanism of bilateral stellectomy. Am J Physiol Heart Circ Physiol 2017;312:H392–H405. 28011590 10.1152/ajpheart.00644.2016 PMC5402012

[33] Millar CK, Kralios FA, Lux RL. Correlation between refractory periods and activation-recovery intervals from electrograms: effects of rate and adrenergic interventions. Circulation 1985;72:1372–1379. 4064279 10.1161/01.cir.72.6.1372

[34] Haws CW, Lux RL. Correlation between in vivo transmembrane action potential durations and activation-recovery intervals from electrograms. Effects of interventions that alter repolarization time. Circulation 1990;81:281–288. 2297832 10.1161/01.cir.81.1.281

[35] Kluge N, Dacey M, Hadaya J, Shivkumar K, Chan SA, Ardell JL, Smith C. Rapid measurement of cardiac neuropeptide dynamics by capacitive immunoprobe in the porcine heart. Am J Physiol Heart Circ Physiol 2021;320:H66–H76. 33095651 10.1152/ajpheart.00674.2020 PMC7847069

[36] Kluge N, Chan SA, Ardell JL, Smith C. Time-resolved in vivo measurement of neuropeptide dynamics by capacitive immunoprobe in porcine heart. J Vis Exp 2022; doi:10.3791/63926 35665743

[37] van Weperen VYH, Hoang JD, Jani NR, Khaky A, Herring N, Smith C, Vaseghi M. Circulating noradrenaline leads to release of neuropeptide Y from cardiac sympathetic nerve terminals via activation of β-adrenergic receptors. J Physiol 2024;603:1911–1921. 38352977 10.1113/JP285945 PMC11322424

[38] Löffelholz K, Muscholl E. A muscarinic inhibition of the noradrenaline release evoked by postganglionic sympathetic nerve stimulation. Naunyn Schmiedebergs Arch Pharmakol 1969;265:1–15. 4254218 10.1007/BF01417206

[39] Löffelholz K, Muscholl E. Inhibition by parasympathetic nerve stimulation of the release of the adrenergic transmitter. Naunyn Schmiedebergs Arch Pharmakol 1970;267:181–184. 4249341 10.1007/BF00999400

[40] Habecker BA, Wang H, Nathanson NM. Multiple second-messenger pathways mediate agonist regulation of muscarinic receptor mRNA expression. Biochemistry 1993;32:4986–4990. 8388252 10.1021/bi00070a003

[41] Caulfield MP . Muscarinic receptors–characterization, coupling and function. Pharmacol Ther 1993;58:319–379. 7504306 10.1016/0163-7258(93)90027-b

[42] Sassi Y, Ahles A, Truong DJ, Baqi Y, Lee SY, Husse B, Hulot JS, Foinquinos A, Thum T, Müller CE, Dendorfer A, Laggerbauer B, Engelhardt S. Cardiac myocyte-secreted cAMP exerts paracrine action via adenosine receptor activation. J Clin Invest 2014;124:5385–5397. 25401477 10.1172/JCI74349 PMC4297204

[43] Yang Q, Sumner AD, Puhl HL, Ruiz-Velasco V. M(1) and M(2) muscarinic acetylcholine receptor subtypes mediate Ca(2+) channel current inhibition in rat sympathetic stellate ganglion neurons. J Neurophysiol 2006;96:2479–2487. 17005606 10.1152/jn.00093.2006

[44] Ajijola OA, Chatterjee NA, Gonzales MJ, Gornbein J, Liu K, Li D, Paterson DJ, Shivkumar K, Singh JP, Herring N. Coronary sinus neuropeptide Y levels and adverse outcomes in patients with stable chronic heart failure. JAMA Cardiol 2020;5:318–325. 31876927 10.1001/jamacardio.2019.4717 PMC6990798

[45] Hulting J, Sollevi A, Ullman B, Franco-Cereceda A, Lundberg JM. Plasma neuropeptide Y on admission to a coronary care unit: raised levels in patients with left heart failure. Cardiovasc Res 1990;24:102–108. 2328516 10.1093/cvr/24.2.102

[46] Maisel AS, Scott NA, Motulsky HJ, Michel MC, Boublik JH, Rivier JE, Ziegler M, Allen RS, Brown MR. Elevation of plasma neuropeptide Y levels in congestive heart failure. Am J Med 1989;86:43–48. 2910096 10.1016/0002-9343(89)90228-3

[47] Ullman B, Hulting J, Lundberg JM. Prognostic value of plasma neuropeptide-Y in coronary care unit patients with and without acute myocardial infarction. Eur Heart J 1994;15:454–461. 8070470 10.1093/oxfordjournals.eurheartj.a060526

[48] Shanks J, Thomson S, Ramchandra R. Neural control of coronary artery blood flow by non-adrenergic and non-cholinergic mechanisms. Exp Physiol 2023;109:2011–2016. 37029787 10.1113/EP090917 PMC11607609

[49] Shanks J, Pachen M, Chang JWH, George B, Ramchandra R. Cardiac vagal nerve activity increases during exercise to enhance coronary blood flow. Circ Res 2023;133:559–571. 37641938 10.1161/CIRCRESAHA.123.323017

[50] Rolf U, Sebastian M, Torun N, Susanne N, Jonas E, Lars E. Neuropeptide Y Y1 and neuropeptide Y Y2 receptors in human cardiovascular tissues. Peptides 2002;23:927–934. 12084524 10.1016/s0196-9781(02)00003-7

[51] McDermott BJ, Millar BC, Dolan FM, Bell D, Balasubramaniam A. Evidence for Y1 and Y2 subtypes of neuropeptide Y receptors linked to opposing postjunctional effects observed in rat cardiac myocytes. Eur J Pharmacol 1997;336:257–265. 9384241 10.1016/s0014-2999(97)01258-2

[52] Vanoli E, De Ferrari GM, Stramba-Badiale M, Hull SS Jr, Foreman RD, Schwartz PJ. Vagal stimulation and prevention of sudden death in conscious dogs with a healed myocardial infarction. Circ Res 1991;68:1471–1481. 2019002 10.1161/01.res.68.5.1471

[53] Li M, Zheng C, Sato T, Kawada T, Sugimachi M, Sunagawa K. Vagal nerve stimulation markedly improves long-term survival after chronic heart failure in rats. Circulation 2004;109:120–124. 14662714 10.1161/01.CIR.0000105721.71640.DA

[54] Gold MR, Van Veldhuisen DJ, Hauptman PJ, Borggrefe M, Kubo SH, Lieberman RA, Milasinovic G, Berman BJ, Djordjevic S, Neelagaru S, Schwartz PJ, Starling RC, Mann DL. Vagus nerve stimulation for the treatment of heart failure: the INOVATE-HF trial. J Am Coll Cardiol 2016;68:149–158. 27058909 10.1016/j.jacc.2016.03.525

[55] Dusi V, De Ferrari GM. Vagal stimulation in heart failure. Herz 2021;46:541–549. 34716778 10.1007/s00059-021-05076-5 PMC8642334

[56] De Ferrari GM, Crijns HJ, Borggrefe M, Milasinovic G, Smid J, Zabel M, Gavazzi A, Sanzo A, Dennert R, Kuschyk J, Raspopovic S, Klein H, Swedberg K, Schwartz PJ; CardioFit Multicenter Trial Investigators. Chronic vagus nerve stimulation: a new and promising therapeutic approach for chronic heart failure. Eur Heart J 2011;32:847–855. 21030409 10.1093/eurheartj/ehq391

[57] Premchand RK, Sharma K, Mittal S, Monteiro R, Dixit S, Libbus I, DiCarlo LA, Ardell JL, Rector TS, Amurthur B, KenKnight BH, Anand IS. Autonomic regulation therapy via left or right cervical vagus nerve stimulation in patients with chronic heart failure: results of the ANTHEM-HF trial. J Card Fail 2014;20:808–816. 25187002 10.1016/j.cardfail.2014.08.009

[58] Carlson MD, Geha AS, Hsu J, Martin PJ, Levy MN, Jacobs G, Waldo AL. Selective stimulation of parasympathetic nerve fibers to the human sinoatrial node. Circulation 1992;85:1311–1317. 1555275 10.1161/01.cir.85.4.1311

[59] Xi Y, Wu G, Ai T, Cheng N, Kalisnik JM, Sun J, Abbasi S, Yang D, Fan C, Yuan X, Wang S, Elayda M, Gregoric ID, Kantharia BK, Lin SF, Cheng J. Ionic mechanisms underlying the effects of vasoactive intestinal polypeptide on canine atrial myocardium. Circ Arrhythm Electrophysiol 2013;6:976–983. 24046327 10.1161/CIRCEP.113.000518

[60] Feliciano L, Henning RJ. Vagal nerve stimulation releases vasoactive intestinal peptide which significantly increases coronary artery blood flow. Cardiovasc Res 1998;40:45–55. 9876316 10.1016/s0008-6363(98)00122-9

[61] Langer SZ . Presynaptic regulation of catecholamine release. Biochem Pharmacol 1974;23:1793–1800. 4617579 10.1016/0006-2952(74)90187-7

[62] Marc B, Frank W, Roland J, Kerstin H, Carsten A, Stefan N, Martin JL, Lutz H. Feedback inhibition of catecholamine release by two different α2-adrenoceptor subtypes prevents progression of heart failure. Circulation 2002;106:2491–2496. 12417548 10.1161/01.cir.0000036600.39600.66

[63] Skovsted P, Sapthavichaikul S. The effects of isoflurane on arterial pressure, pulse rate, autonomic nervous activity, and barostatic reflexes. Canadian Anaesthetists’ Society Journal 1977;24:304–314. 871935 10.1007/BF03005103

[64] Seagard JL, Elegbe EO, Hopp FA, Bosnjak ZJ, von Colditz JH, Kalbfleisch JH, Kampine JP. Effects of isoflurane on the baroreceptor reflex. Anesthesiology 1983;59:511–520. 6650907 10.1097/00000542-198312000-00005

[65] Benson GJ, Thurmon JC. Anesthesia and analgesia for swine. In: Kohn DF, Wixson SK, White WJ, Benson GJ (eds.), Anesthesia and Analgesia in Laboratory Animals. San Diego: Academic Press; 1997:225–242.

[66] Silverman J, Muir WWI. A review of laboratory animal anesthesia with chloral hydrate and chloralose. Lab Anim Sci 1993;43:210–216. 8355479

[67] Flecknell PA . Laboratory Animal Anaesthesia. London, UK: Academic Press; 1996.

