# Supplementary material for: Sympathovagal crosstalk: Y2-receptor blockade enhances vagal effects which in turn reduce NPY levels via muscarinic receptor activation
Source: Cardiovasc Res. 2025 Oct 6;121(14):2189–203. doi: 10.1093/cvr/cvaf180 (PMC12638732; doi:10.1093/cvr/cvaf180)
Supplement: cvaf180_Supplementary_Data [file cvaf180_supplementary_data.docx]

**Supplemental Figure 1.**


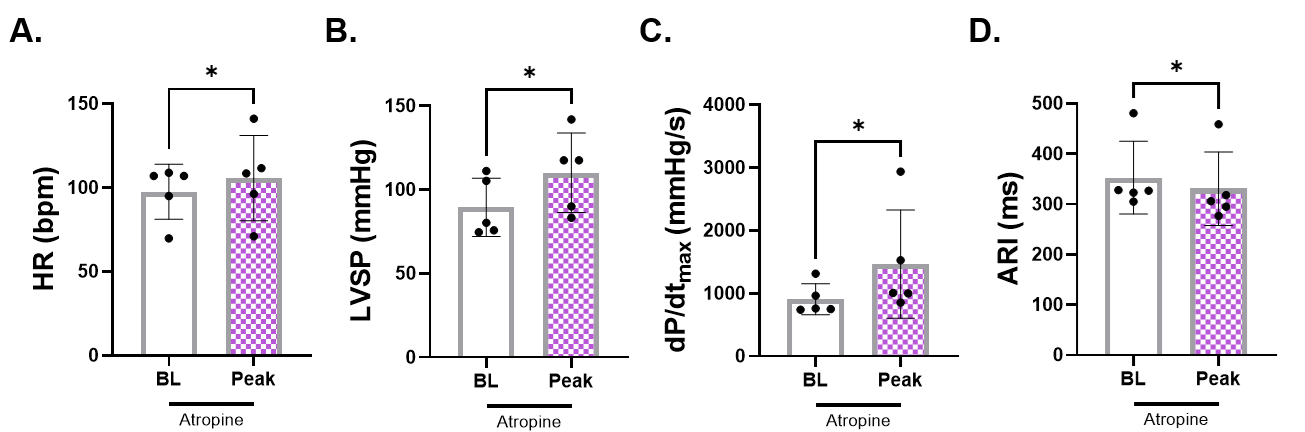


**Supplemental Figure 1. Acute hemodynamic and electrophysiological effects of atropine. (A-C)** Atropine significantly increased HR, LVSP, and dP/dtmax from pre-stimulation (baseline) values. **(D)** ARI was significantly shortened from pre-stimulation (baseline) values. *P < 0.05, n = 5 for all comparisons.

**Supplemental Figure 2.**


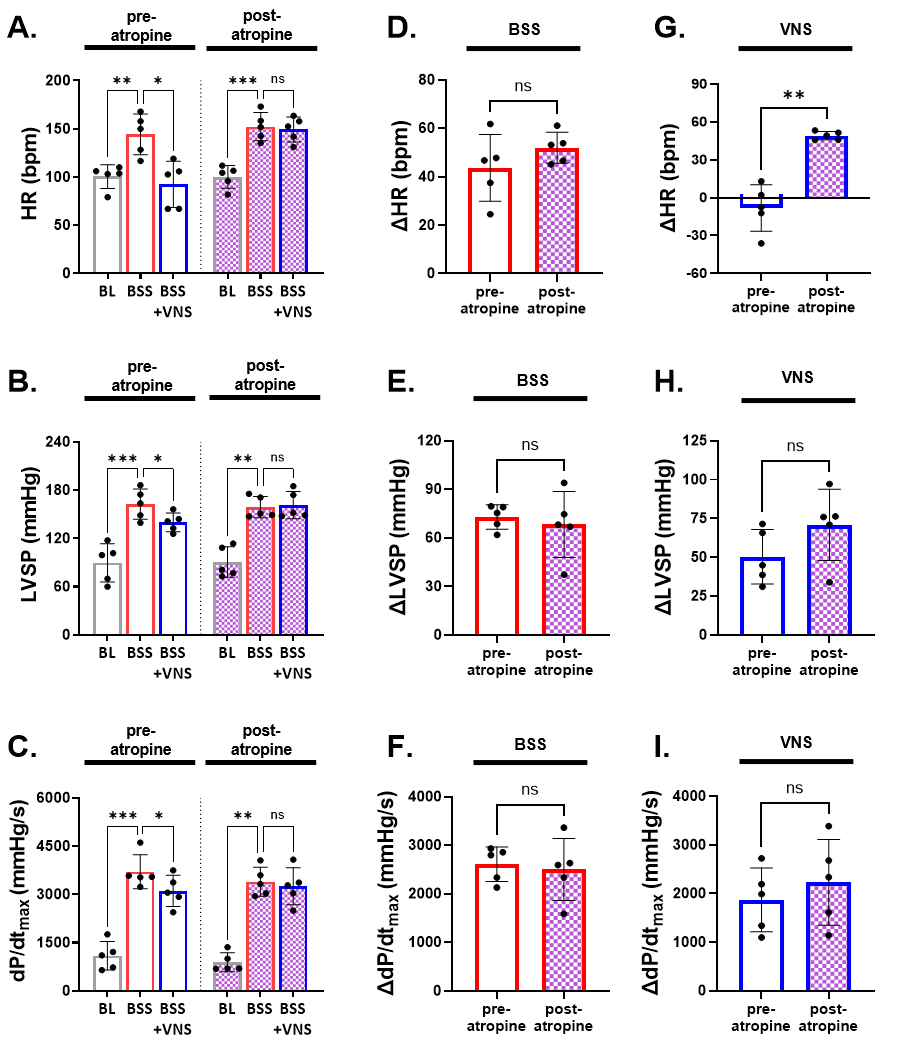


**Supplemental Figure 2. Hemodynamic effects of BSS+VNS before and after administration of atropine.** BSS significantly increased mean **(A)** HR, **(B)** LVSP, and **(C)** dP/dtmax from pre-stimulation (baseline) values. As expected, atropine blocked the hemodynamic effects of VNS during BSS. **(D-F)** Hemodynamic effects of BSS alone were not significantly different pre- vs. post-atropine. *P < 0.05, **P < 0.01, ***P < 0.001, n = 5 for all comparisons. P ≤ 0.05 was considered statistically significant, ns = not statistically significant.

**Supplemental Figure 3.**

**
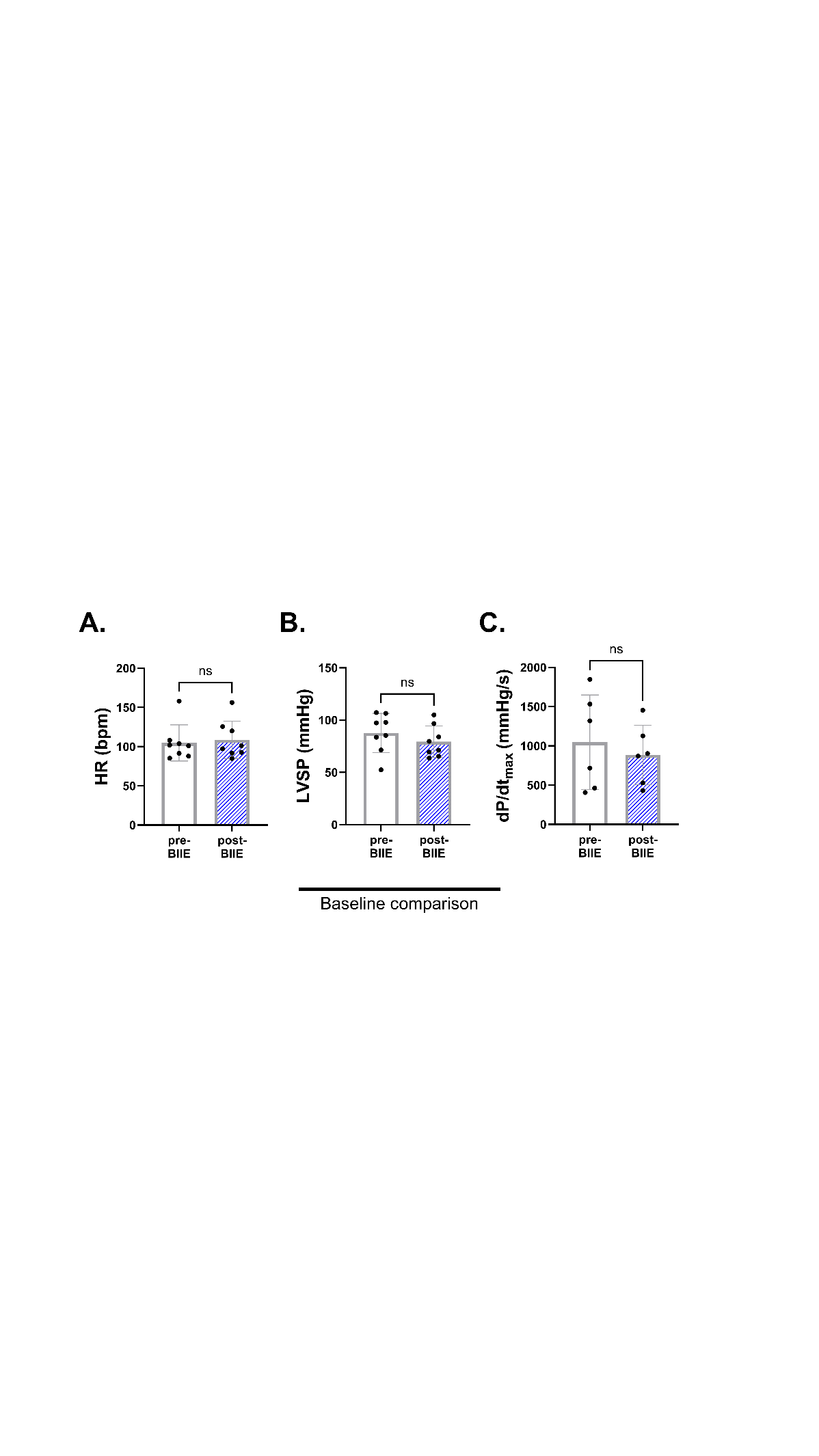
**

**Supplemental Figure 3. Evaluation of hemodynamic parameters before and after administration of BIIE0246**. No significant difference was observed in **(A)** HR, **(B)** LVSP, and **(C)** dP/dt_max_ before vs. after the administration of BIIE0246 (BIIE). Measurements were obtained at 20 min after BIIE0246 administration (prior to any autonomic nerve stimulations) and compared to pre-BIIE2046 hemodynamic parameters. P ≤ 0.05 was considered statistically significant; ns = not statistically significant, n = 8 for HR and LVSP, n = 6 for dP/dt_max_.
